# Cancer Immunotherapy in Combination with Radiotherapy and/or Chemotherapy: Mechanisms and Clinical Therapy

**DOI:** 10.1002/mco2.70346

**Published:** 2025-08-31

**Authors:** Xinmin Wang, Jing Jing

**Affiliations:** ^1^ Institute of Breast Health Medicine West China Hospital, Sichuan University Chengdu Chengdu Sichuan China; ^2^ Department of General Surgery West China Hospital, Sichuan University Chengdu Sichuan China

**Keywords:** immunomodulatory effects, immunotherapy, radiotherapy, tumor, Chemotherapy

## Abstract

Since the United States Food and Drug Administration approved the first immune checkpoint inhibitor ipilimumab for metastatic melanoma in 2011, ICIs have been approved for a range of cancers and significantly improving treatment outcomes. However, the objective response rate of ICI monotherapy remains modest (10–40%), with clinical benefit observed in only 15–20% of patients. The limited efficacy of ICIs in many patients is often attributed to poorly immunogenic (“cold”) tumors. Radiotherapy and chemotherapy exhibit immunomodulatory properties that can enhance tumor immunogenicity. These effects provide a rationale for combining ICIs with conventional therapies. Current research lacks systematic synthesis and consistent clinical evidence on the immunomodulatory effects of radio/chemotherapy, and the optimal selection and sequencing of radio/chemotherapy with immunotherapy remain unclear, limiting the optimization of combination strategies with immunotherapy. This review outlines the current landscape of cancer immunotherapy and elucidates the immunomodulatory effects of radiotherapy and chemotherapy that form the basis for combination strategies. It further summarizes clinical advances in combined modalities and discusses associated toxicities, management approaches, and potential predictive biomarkers. This review provides a comprehensive framework for understanding and optimizing radio/chemo‐immunotherapy by integrating mechanistic insights with clinical evidence to guide future personalized cancer treatment strategies.

## Graphical abstract

Immunotherapy has revolutionized the clinical management of many malignancies. However, the presence of immune “cold” tumors limits objective response rates, highlighting the need for strategies to enhance the efficacy of immunotherapy. Radiotherapy has gained attention for its immunostimulatory abilities, particularly the “abscopal effect.” Chemotherapy, owing to its broad practicality, is also a promising partner for immunotherapy. Nonetheless, current clinical studies on combined radio/chemo‐immunotherapy show controversial results regarding efficacy and safety. These inconsistencies may stem from the traditional focus of radiotherapy and chemotherapy on maximizing cancer cell destruction and minimizing toxicity to healthy tissue, while overlooking their potential immunostimulatory effects. This highlights the need to adapt radio‐ and chemotherapy protocols to better align with immunotherapy. In this review, we discuss the immune effects induced by radio‐ and chemotherapy and the underlying mechanisms, including changes in the immune microenvironment caused by different doses and fractionation schemes, the optimal sequencing of immunotherapy relative to radio/chemotherapy, and safety considerations for these combined approaches.

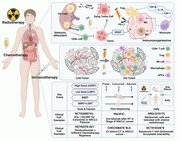

## 1 Introduction

In 2011, ipilimumab, an immune checkpoint inhibitor (ICI) targeting cytotoxic T‐lymphocyte antigen 4 (CTLA‐4), was approved for the treatment of metastatic melanoma (MM) [1]. Since then, a growing number of ICIs have received United States Food and Drug Administration (US FDA) approval for various malignancies [2, 3, 4, 5], bringing significant improvements in cancer treatment outcomes [6, 7]. However, challenges remain. Studies show that the objective response rate (ORR) for ICIs monotherapy remains low, typically between 10 and 40% [8, 9, 10, 11], with only 15–20% of patients experiencing clinical benefits [8, 12]. Although some patients experience durable responses, many patients are accompanied with “cold” tumors [13]—those with limited immune infiltration, remain resistant to ICIs, limiting treatment efficacy [14, 15]. Clinical studies [16] have shown that treatment with CTLA‐4 inhibitors yields response rates of approximately 15%, while those achieved with programmed cell death ‐ protein1/ligand 1 (PD‐1/PD‐L1) inhibitors rarely exceed 40%. Consequently, enhancing the response rate and overall effectiveness of immunotherapy (IT) to improve patient prognosis remains urgent needs [17]. Recent years, the strategy of combining IT with other cancer treatments, particularly radiotherapy (RT) and chemotherapy (CT) has attracted widespread attention [13, 18, 19, 20].

RT is widely used for both radical and palliative treatment in variety of malignant tumors [21], with around 50–60% of cancer patients receiving it [22, 23]. CT is also considered a classical systemic treatment for tumors. Due to its practicality, it is widely applied in the management of various solid tumors and hematological malignancies. While numerous studies [24, 25, 26, 27, 28, 29] highlight RT and CT's immunosuppressive effects, increasing preclinical and clinical evidence suggests it also has immunostimulatory effects [30, 31].

Despite there are promising preclinical findings, the clinical outcomes of combining RT/CT and IT remain variable. The ASTEROID study [32] and KEYNOTE189 [33] support the feasibility of combining RT/CT with IT. However, there is still no consensus regarding the immunomodulatory effects induced by different doses or fractionation schemes of RT and/or CT, as well as the potential these effects hold for combination with IT. Moreover, existing clinical findings show substantial inconsistency. The study by Schoenfeld et al. [34] did not observe significant efficacy from the combination of RT and IT. Similarly, the therapeutic benefits of CT‐IT remain to be further validated in the study by Rajani et al. [35]. In addition, the toxicity of RT/CT combined with IT remains a major concern. To date, there is still a lack of comprehensive reviews that systematically summarize the immunomodulatory mechanisms of RT and CT, clarify the conditions that are more favorable for the success of subsequent IT, and integrate these insights with clinical studies on combination strategies.

Building on the above, this review first provides a brief overview of the current landscape of IT, with a particular focus on ICIs. It then examines the rationale and mechanisms of combining IT with RT or CT, highlighting both their immune‐activating and immunosuppressive effects. We further analyze how these therapies may synergize with IT, outline their roles within the tumor immune microenvironment, and summarize current clinical progress and key challenges in combination strategies. This section includes discussions on dosing, fractionation, timing, and sequencing. Finally, we explore the potential toxicities of combined RT‐ or CT‐IT, including underlying mechanisms and possible predictive biomarkers. By integrating mechanistic understanding with clinical evidence, this review aims to provide a theoretical basis and research direction for optimizing and personalizing future combination treatments.

## 2 Overview of Cancer IT Approaches

In this section, we provide an overview of the five common types of IT—particularly ICIs—and discuss their roles and potential in combination strategies.

### 2.1 Cytokine Therapies

The earliest cytokine therapies approved for clinical cancer treatment included interleukin‐2 (IL‐2) and interferon‐α (IFN‐α) [36]. Initially identified as a T‐cell growth factor, IL‐2 promotes the proliferation and differentiation of CD4⁺ and CD8⁺ T cells while enhancing natural killer (NK) cell activity and B‐cell antibody production, thereby enhancing both the activation and termination of immune responses [37, 38, 39]. In 1992, a landmark clinical trial by Fyfe et al. [40] involving 255 patients with metastatic renal cell carcinoma (mRCC), high‐dose IL‐2 elicited an ORR of 14%, leading to its US FDA approval as the first cytokine‐based IT for metastatic cancer. Subsequent studies demonstrated its efficacy in MM, with durable responses observed in a subset of patients [41]. IFN‐α was the first cytokine approved for cancer therapy, initially used to treat hairy cell leukemia. In a study by Kirkwood et al. [42], high‐dose IFN‐α as adjuvant therapy significantly prolonged both relapse‐free survival and overall survival (OS) in high‐risk melanoma patients following surgery, becoming the first cytokine therapy to demonstrate survival benefits for this population in randomized controlled trials (RCTs). Although IL‐2 and IFN‐α therapies have validated the potential of cytokines to enhance antitumor immunity, their systemic toxicity remains a major limitation for clinical application. High‐dose IL‐2 frequently induces capillary leak syndrome and severe hemodynamic instability, while IFN‐α is associated with broad‐spectrum adverse effects including fatigue, depression, and myelosuppression [43, 44]. Consequently, recent research has focused on optimizing cytokine formulations [45], exploring combination strategies with chemotherapeutic agents (NCT00994643) [46], and integrating them with adoptive cell therapy (ACT) [47] or ICI to improve efficacy while enhancing tolerability [48, 49]. RT can also be combined with it, and numerous clinical trials are currently underway to investigate the synergistic potential of this approach [50, 51, 52].

### 2.2 Oncolytic Virus Therapies

Oncolytic virus (OV) therapy is an emerging modality in cancer IT that utilizes naturally occurring or genetically engineered viruses to selectively infect and lyse tumor cells. In addition to direct oncolysis, OV therapy stimulates the host immune system to elicit durable antitumor immune responses [53, 54, 55, 56]. Unlike conventional therapies, OVs offer the dual advantage of achieving localized tumor destruction and systemic immune activation, demonstrating unique potential in treating certain immunologically “cold” tumors. In 2015, talimogene laherparepvec (T‐VEC), a modified herpes simplex virus type 1, became the first OV product globally approved by the US FDA for unresectable melanoma, marking a pivotal clinical breakthrough in the field [57]. Notably, H101 had previously emerged as the first OV approved for cancer therapy in China in 2005. A prospective, randomized controlled clinical trial [58] demonstrated that intratumoral H101 injection combined with CT achieved an ORR of 72.7% in patients with head/neck or esophageal squamous cell carcinoma, compared with 40.3% with CT alone, while maintaining favorable tolerability. The approval of H101 established a critical clinical precedent for OV, particularly validating its efficacy and safety in Eastern populations, the mechanism by which OVs eliminate tumor cells is distinct from that of conventional anticancer therapies. Notably, existing studies have shown minimal overlap in toxicity profiles when OVs are administered in combination with other modalities [59, 60]. This low redundancy in adverse effects supports the rationale for integrating OVs with a broad spectrum of treatments, including RT and CT, thereby providing a solid foundation for the development of rational viral design and combinatorial therapeutic strategies [61, 62].

### 2.3 Cancer Vaccines

Vaccines designed to prevent infectious diseases stand as one of the greatest medical advances of the 20th century. However, the concept that antigens can elicit immunogenicity by providing sufficient immune stimulation is no longer confined to infectious diseases [63]. This principle traces back to the late 19th century work of William B. Coley, who observed tumor regression in some sarcoma patients treated with a mixture of Streptococcus and Serratia toxins (Coley's toxins) [64]. By the 1980s, early cancer vaccines emerged, exemplified by Herbert et al.’s study [65] demonstrating that colorectal cancer (CRC) patients receiving autologous tumor cells combined with BCG vaccine had a 15% recurrence rate (three out of 20) with no deaths, compared with 45% recurrence (nine out of 20) and 20% mortality (four out of 20) in the control group. Cancer vaccines function by activating and expanding tumor‐specific T cells (primarily CD8⁺ cytotoxic T lymphocytes [CTLs]), thereby enhancing tumor recognition and elimination while modulating the tumor immune microenvironment to restore immune surveillance capabilities [66, 67]. A key priority in cancer IT has been the identification of optimal antigens targeting specific tumor types [68]. Tumor antigens can be classified into tumor‐associated antigens (TAAs) and tumor‐specific antigens (TSAs). TAAs are broadly expressed in tumors and some normal tissues [69] (e.g., human telomerase reverse transcriptase, Human Epidermal Growth Factor Receptor 2[HER2]), whereas TSAs arise from tumor‐specific mutations and are exclusively expressed in malignant cells [70] (e.g., human papillomavirus [HPV] antigens). In the 1990s, van der Bruggen et al. [66] pioneered the identification of the first human TSA—melanoma antigen MAGE‐A1—through T‐cell clone screening, establishing antigen‐specific targeting as a milestone in cancer vaccine design. This breakthrough translated into a phase II trial evaluating recombinant MAGE‐A3 protein combined with immunostimulants in MAGE‐A3‐positive stage IB–II non‐small cell lung cancer (NSCLC) patients postresection. Results demonstrated a lower recurrence rate in the treatment group (*n* = 122; 35 vs. 43% placebo) and universal humoral immune responses against MAGE‐A3 among vaccinated patients [71]. Although intracellular TAAs can be presented by HLA molecules for recognition, the absence of costimulatory signals and immunosuppressive microenvironments often render T cells anergic [63]. Thus, effective antigen presentation requires fully functional antigen‐presenting cells (APCs), particularly dendritic cells (DCs) [72]. Sipuleucel‐T (Provenge; Dendreon), a DC‐based autologous prostate cancer vaccine, became the first US FDA‐approved therapeutic cancer vaccine in humans [73]. These collective advances underscore the translational potential and clinical significance of tumor vaccines in oncology therapeutics.

### 2.4 Adoptive Cell Transfer

Adoptive Cell Transfer (ACT) represents a therapeutic strategy involving the ex vivo activation, expansion, or genetic modification of autologous or allogeneic immune cells, followed by reinfusion into patients to enhance antitumor immunity [74]. The foundation of ACT was established in 1955 by Mitchison [75], who demonstrated that only activated lymphocytes from tumor‐draining lymph nodes could effectively transfer antitumor immunity. Subsequent work by Fefer [76] and Rosenberg et al. [77] in murine sarcoma virus‐induced tumor models provided critical proof‐of‐concept: activated lymphocytes or their derivatives mediated antitumor effects even in immunocompromised hosts or those with established tumor burden, underscoring ACTs therapeutic potential. The field advanced further when the team of Rosenberg refined this approach using tumor‐infiltrating lymphocytes (TILs), showing that adoptive transfer of IL‐2‐expanded TILs significantly improved efficacy [78, 79]. These pivotal studies laid the mechanistic groundwork for TIL‐based therapies in advanced human cancers. To overcome the limitations of TIL therapy and expand the applicability of ACT, researchers developed methods to genetically engineer peripheral T cells by introducing genes encoding tumor‐targeting receptors. This strategy enables artificial redirection of T cell specificity through transfection of either conventional αβ T cell receptors (TCRs) or chimeric antigen receptor (CAR) genes [80]. CAR T‐cell therapy was first conceptualized by Gross et al. [81] in the late 1980s. The initial design fused a single‐chain variable fragment (scFv) derived from monoclonal antibodies with T‐cell activation domains (e.g., CD3ζ or FcεRIγ), enabling MHC‐independent tumor antigen recognition. While demonstrating antitumor activity in various animal models [82, 83], an early clinical trial by Lamers et al. [84] showed that engineered T cells expressing scFv(G250) induced antigen‐specific immune responses but failed to halt disease progression in metastatic clear cell renal carcinoma (RCC) patients. Subsequent advancements came from Sadelain et al. [85,86], who pioneered a second‐generation CAR incorporating both CD3ζ and CD28 signaling domains, significantly enhancing T‐cell proliferation, IL‐2 secretion, and cytotoxic function in vitro and in vivo [87]. Further innovation by Campana's team introduced the 4‐1BB costimulatory domain into CAR constructs [88, 89, 90]. Recently, dual‐targeting CARs (e.g., CD19/CD20) have shown promising safety and preliminary efficacy in early‐phase trials, offering a novel therapeutic approach for B‐cell malignancies [91].

### 2.5 Immune Checkpoint Inhibitors

**FIGURE 1:**
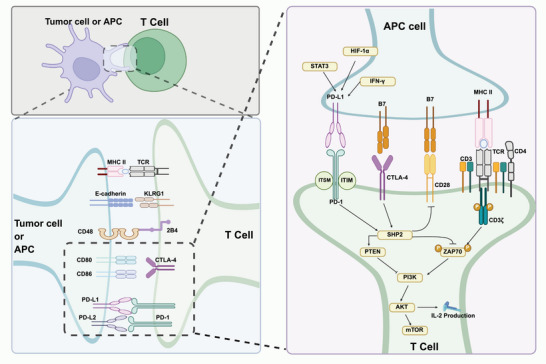
Immune checkpoint‐mediated inhibition of T cell antitumor activity. Top left: Overview of the interactions between tumor cells or APCs and T cells, highlighting key immune checkpoints that suppress T cell activation in the tumor microenvironment. Bottom left: Zoomed‐in view showing major inhibitory receptors on T cells—CTLA‐4, PD‐1, 2B4, KLRG1—and their ligands on APCs or tumor cells, including CD80, CD86, PD‐L1, PD‐L2, MHC‐II, and others. Right panel: Downstream signaling of CTLA‐4 and PD‐1. CTLA‐4 competes with CD28 for CD80/CD86 binding, recruits SHP2, and inhibits the PI3K/AKT/mTOR pathway, reducing IL‐2 production. PD‐1 binds PD‐L1/PD‐L2 and similarly recruits SHP2, which dephosphorylates ZAP70 and other TCR‐proximal signaling components, thereby suppressing TCR signaling and impairing T cell activation. HIF‐1α, Hypoxia‐Inducible Factor 1‐alpha; ITIM, Immunoreceptor Tyrosine‐based Inhibitory Motif; ITSM, Immunoreceptor Tyrosine‐based Switch Motif; KLRG1, Killer Cell Lectin Like Receptor G1; PTEN, Phosphatase and Tensin Homolog; SHP2, Src Homology 2 Domain‐containing Phosphatase 2; STAT3, Signal Transducer and Activator of Transcription 3; ZAP70, Zeta‐chain‐associated Protein Kinase 70.

ICIs have revolutionized cancer IT by targeting key regulators of immune tolerance, notably CTLA‐4, PD‐1, and PD‐L1 [92, 93]. These molecules are often exploited by the tumor microenvironment (TME) to suppress T cell activity and promote immune escape [94, 95]. CTLA‐4 is upregulated during the priming phase of T cell activation and inhibits costimulatory signaling by competitively binding CD80/CD86 with higher affinity than CD28, thereby impairing naive T cell activation [96, 97]. Ipilimumab, the first approved monoclonal antibody targeting CTLA‐4, has demonstrated significant survival benefit in advanced melanoma, attributed to both enhanced T cell activation and functional suppression of regulatory T (Treg) cells [98]. In contrast, the PD‐1/PD‐L1 axis operates predominantly during the effector phase. PD‐1 is expressed on chronically stimulated T cells [99], and upon engagement with PD‐L1—expressed on tumor cells, endothelial cells, DCs, and other immune subsets—it recruits SHP‐2 phosphatase, leading to dephosphorylation of key TCR signaling intermediates including ZAP70, PI3K/Akt, and mTOR [100]. This cascade culminates in reduced IL‐2 production, and the induction of T cell exhaustion. Studies by Parsa et al. [101] have demonstrated that both type I and type II IFNs can upregulate PD‐L1 expression, establishing a state of adaptive immune resistance. Notably, the phase III ATTRACTION‐3 trial demonstrated that nivolumab significantly prolonged OS in patients with advanced esophageal squamous cell carcinoma (median OS: 10.9 vs. 8.4 months; hazard ratio [HR] = 0.77, *p* = 0.019), with a favorable safety profile relative to CT [102]. Nonetheless, primary and acquired resistance remain major obstacles. Contributing mechanisms include Treg cell expansion, enrichment of myeloid‐derived suppressor cells (MDSCs), impaired immune signaling, antigen loss, and aberrant tumor vasculature. Sustained activation of STAT3 and HIF‐1α has been implicated in PD‐L1 upregulation and immune suppression, while PI3K/Akt signaling not only promotes tumor proliferation but also disrupts antigen presentation and T cell infiltration [103, 104] (Figure 1). To counteract these immune evasion mechanisms, combinatorial strategies have been proposed. RT induces immunogenic cell death (ICD) and releases damage‐associated molecular patterns (DAMPs), whereas CT can remodel the TME and enhance immune cell infiltration, thereby potentiating ICI‐mediated antitumor responses. Given that ICIs constitute the principal class of immunotherapeutic agents currently approved by the US FDA and widely adopted in clinical oncology, subsequent sections of this review will focus primarily on ICI‐combined strategies [105].

## 3 Rationale and Mechanism of Combining IT with Traditional Therapies

In this section, we mainly discuss the rationale and mechanisms of combining IT with RT or CT, emphasizing both their immune‐activating and immunosuppressive effects.

### 3.1 RT and Abscopal Effect

RT is a widely used treatment that destroys cancer cells and reduces tumor size by damaging DNA with high‐energy radiation, making it essential in cancer care [106]. Its primary mechanism involves breaking chemical bonds between DNA bases and intracellular components, leading to mitotic catastrophe in tumor cells, which ultimately results in tumor cell death [107, 108]. Beyond direct cell destruction, RT has been shown to stimulate systemic immune responses [109]. The abscopal effect, first described by Mole [110] in 1953, is a hallmark of RT‐induced immunomodulation, where irradiation of a single tumor site is followed by regression of metastatic lesions outside the radiation field. This phenomenon reflects a systemic antitumor immune response associated with radiation, resulting from a balance between RT‐induced immune activation and immunosuppressive mechanisms [111].

### 3.2 Immunostimulant Effects of RT

#### 3.2.1 Antigenicity

When RT is applied to tumor tissue, tumor cells often experience cellular stress and release TAAs into the surrounding environment [112, 113]. These antigens are captured by APCs, particularly DCs. APCs present TAAs to CD8+ T cells via MHC class I molecules, initiating a specific antitumor immune response and promoting tumor reduction [114]. Additionally, RT can also enhance the expression of previously underexpressed antigens [115]. Research by Reits et al. [116] demonstrated a radiation dose‐dependent increase in the surface expression of MHC class I molecules, which typically occurs 18 h after irradiation and can persist for approximately 10 days. This upregulation may arise from several mechanisms [116]. First, RT promotes the unfolding and degradation of proteins in tumor cells, especially those mutated by nonsynonymous mutations [117]. This degradation increases the diversity of the peptide library, leading to the production of new TAAs. Second, RT induces transient expression of MHC/peptide complexes on tumor stromal cells by activating the mTOR pathway, these newly generated peptides bind to MHC I and are presented on the surface of tumor cells, significantly enhancing their immunogenicity [117, 118]. This enhancement improves the ability of antigen recognition, binding to T cells and promoting the release of cytokines such as IFN‐γ, which is mediated through pathways involving protein kinase C, nuclear factor kappa B, NFAT, and Ras/Erk [119, 120].

#### 3.2.2 Induction of ICD

Induction of ICD in tumor cells is regarded as a key mechanism for RT to stimulate antitumor immune responses [108, 121, 122]. Following ICD, tumor cells release DAMPs, including calreticulin (CRT), high mobility group protein 1 (HMGB1), along with other factors such as adenosine‐5′‐triphosphate (ATP), and heat shock proteins (HSPs) [123, 124, 125]. In normal cells, CRT is predominantly located in the endoplasmic reticulum (ER), where it functions as a calcium‐binding protein involving in calcium storage and protein folding, remaining largely hidden from the cell surface [126]. However, after induction by RT, the ER‐resident protein complex CRT/ERp57 translocates to the cell membrane, acting as a “eat me” signal, and binds to the CD91 receptor on APCs [127], enhancing the uptake of TAAs and initiating the antigen presentation process. Concurrently, triggering the release of proinflammatory cytokines such as tumor necrosis factor‐alpha (TNF‐α) and IL‐6 [128], promoting the phagocytosis and subsequent elimination of tumor cells [129, 130].

HMGB1 is a nuclear protein primarily responsible for regulating gene expression and DNA repair [131, 132, 133]. Following RT, HMGB1 is released from the nucleus into the extracellular space, which is a hallmark of ICD [133]. Once outside the cell, HMGB1 binds to receptors on DCs, such as Toll‐like receptor 4 (TLR4) and receptor for advanced glycation end products, promoting DC maturation and stimulating the release of proinflammatory cytokines like IL‐1β [134]. This process generates a potent immune‐stimulating effect [135, 136].

ATP, released through autophagy as tumor cells undergo cell death, interacts with P2X7 (which activates the NLRP3 inflammasome) and P2RY2 receptors on DCs and macrophages [137]. This interaction further promotes the recruitment of APCs—including DCs, macrophages, and monocytes—each the area where ICD occurs, where they uptake antigens from dead tumor cells and initiate a robust immune response [138, 139].

HSPs are highly conserved proteins known for their cytoprotective and antiapoptotic abilities [140]. In the context of ICD, HSPs play a role in helping APCs reposition to tumor cells and enhancing antitumor immune responses [125]. Notably, HSP70 can interact with the costimulatory molecule CD40, which is primarily expressed on APCs, thereby promoting the activation of CD8+ T cells [141, 142]. HSP70 is also shown to directly activate and stimulate the cytolytic functions of NK cells [143]. HSP90, on the other hand, binds to the CD91 receptor, creating a prophagocytic signal that enhances DC activation and further supports immune cell engagement against the tumor [144].

#### 3.2.3 cGAS–STING

After RT, cells can experience DNA double‐strand breaks (DSBs), which are detected by the cyclic GMP‐AMP synthase (cGAS) protein sensor [122, 145]. This recognition triggers the synthesis of cyclic GMP‐AMP (cGAMP), which serves as a secondary messenger. cGAMP activates and binds to the stimulator of IFN genes (STING), prompting its translocation from the ER to the Golgi apparatus [146, 147]. This activation drives downstream signaling pathways, as STING recruits TANK‐binding kinase 1, which phosphorylates IFN regulatory factor 3 [148]. This cascade leads to the production of proinflammatory cytokines, such as IFN‐1, inducing a robust immune response [149]. Recent studies [146, 150, 151] have also demonstrated that exogenous cGAMP or synthetic STING agonists can enhance the effects of RT in mouse models.

The study by Bao et al. [152] proposed an integrative model demonstrating that the telomerase inhibitor BIBR1532 enhances the activation of the cGAS–STING pathway in response to RT. Mechanistically, BIBR1532 promotes ferroptosis, which leads to the release of mitochondrial DNA (mtDNA), thereby providing an additional stimulus for cGAS–STING activation. This finding expands the conventional understanding that only nuclear DNA damage‐derived double‐stranded DNA serves as a trigger for cGAS recognition. It highlights that mtDNA released upon mitochondrial stress can also function as pathogen‐associated molecular patterns to activate the STING signaling axis. These insights offer a theoretical foundation for developing novel combination strategies integrating RT and IT. Suppression of the cGAS–STING–IFN signaling pathway has been associated with radioresistance. Li et al. [153] demonstrated that PREX2 plays a pivotal role in promoting radioresistance in CRC, partly by inhibiting the cGAS–STING–IFN axis. Wen and Zhang further substantiated the critical role of cGAS–STING pathway suppression in mediating radioresistance. Zhang et al. [154] demonstrated that RT‐induced upregulation of heme oxygenase‐1 (HO‐1) and its catabolites impairs antitumor immunity by disrupting STING oligomerization and its ER‐to‐Golgi translocation, as well as inhibiting the nuclear export of cGAS. These events collectively suppress cGAMP production and downstream IFN‐I signaling. In a complementary study, Wen et al. [155] revealed that radiation activates the STING/IFN‐I axis to induce expression of the m6A reader protein YTHDF1 in DCs, which in turn promotes lysosomal cathepsin‐mediated degradation of STING, attenuating IFN‐I production and impairing cross‐presentation. Both studies identify HO‐1 and YTHDF1 as actionable targets for restoring STING signaling, thereby enhancing the efficacy of RT and RT‐IT and offering new strategies to overcome radioresistance.

**FIGURE 2:**
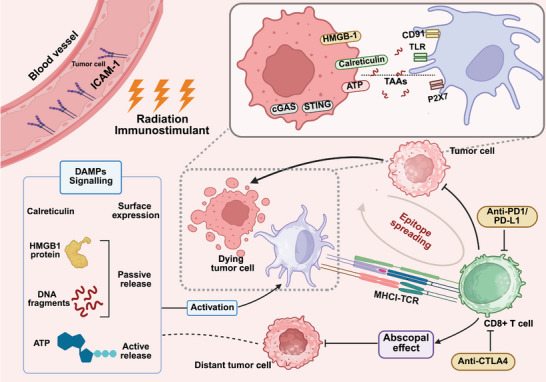
Immunostimulatory effects of radiotherapy. This figure illustrates the mechanisms underlying the immunostimulatory effects of radiotherapy. It includes: (1) The upregulation of new antigens, an increase in TAAs, and enhanced antigen presentation by APCs. (2) The occurrence of ICD, the release of DAMPs, and their interaction with antigen‐presenting cells. (3) The upregulation of major histocompatibility complex (MHC) class I molecules and adhesion molecules (e.g., ICAM‐1).

In summary, RT enhances the presentation of TAAs, increases CD8+ T cell infiltration within the immune microenvironment, and promotes the release of various immune‐related cytokines [156]. Together, these changes transform the TME from “cold” to “hot,” turning it into an immune‐reactive site and thereby boosting the effectiveness of IT [157, 158]. The immunostimulant effects of RT described above are illustrated in Figure 2.

### 3.3 Immunosuppressant Effects of RT

**FIGURE 3:**
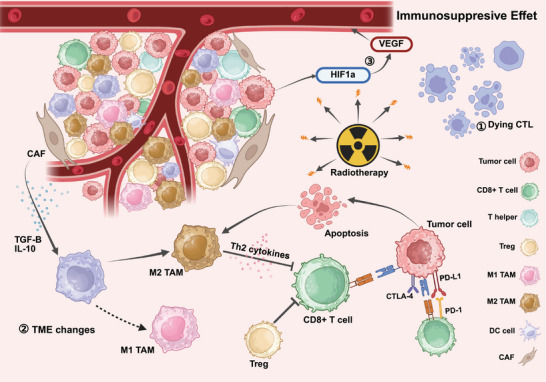
Immunosuppressive effects of radiotherapy. Radiotherapy exerts prominent immunosuppressive effects, manifested in three main aspects: (1) Direct immunosuppressive effects: Radiotherapy induces direct cytotoxicity on lymphoid organs and immune cells. (2) Radiation‐induced changes in the TME lead to the release of immunosuppressive factors from tumor‐associated fibroblasts (CAF), such as transforming growth factor‐beta (TGF‐β) and IL‐10, promoting M2 macrophage polarization, as well as increased infiltration of myeloid‐derived suppressor cells (MDSCs) and regulatory T (Treg) cells. This results in the production of immunosuppressive cytokines and reduced infiltration of CD8+ T cells in the TME. (3) Upregulation of hypoxia‐inducible factor 1 (HIF‐1) and vascular endothelial growth factor (VEGF), contributing to enhanced angiogenesis.

In addition to generating cellular and tissue responses that promote immune‐mediated tumor rejection, RT may also enhance immunosuppressive effects through various mechanisms [114, 159]. These include damaging lymphoid organs and immune cells, intensifying the existing immunosuppressive TME, and inducing the expression of molecules that hinder DC cross‐presentation and/or T cell‐mediated tumor cell killing [160]. The immunosuppressant effects of RT described below are illustrated in Figure 3.

#### 3.3.1 Lymph Node Suppression and Lymphopenia

In recent years, the therapeutic approach to RT has evolved to maximize tumor cell destruction while preserving surrounding normal tissue as much as possible [161]. However, due to the high sensitivity of lymphocytes to radiation, RT can directly impact their survival and function by inducing DNA damage, apoptosis, and cell cycle arrest. Consequently, radiation‐induced lymphopenia (RIL) becomes a common outcome as blood exposure within the irradiated area reduces lymphocyte levels [162]. Studies [163, 164, 165] show that 40–70% of patients receiving RT experience RIL. Although circulating lymphocyte counts often return to baseline relatively quickly posttreatment, research indicates that lymphopenia significantly increases the risk of tumor progression [166]. The edges of directly irradiated areas, including bone marrow or spleen located outside or near the radiation field, are often affected by low‐dose radiation (approximately 5–10% of the tumor dose). This exposure impacts and depletes lymphocytes in these regions [166].

#### 3.3.2 Effects on TME

The TME consists of blood vessels, extracellular matrix, cancer‐associated fibroblasts (CAFs), and various immune cells, including T and B lymphocytes, tumor‐associated macrophages (TAMs), NK cells, and MDSCs [167, 168]. These components play crucial roles in tumor initiation and progression.

#### 3.3.3 Effects on Immunosuppressive Immune Cells

Following RT, a range of immunosuppressive cytokines, such as CSF‐1, CCL2, and CXCL12, are released, which promote the recruitment of Treg cells, MDSCs, TAMs, and other immunosuppressive cells into the tumor tissue [24, 169, 170, 171].

Treg, a subset of CD4+ T cells characterized by the expression of the transcription factor forkhead box P3 (FOXP3). In the TME, Treg cells exert immunosuppressive effects by secreting inhibitory transforming growth factor beta (TGF‐β), IL‐10, and so on, and directly suppressing effector immune cells, including CD8+ T cells and NK cells, thereby inhibiting effector T cell activation [172]. Studies have shown that the number of Treg  increased after local or systemic irradiation, with elevated levels of TGF‐β and IL‐10 observed at various radiation doses [25, 173]. One proposed mechanism underlying the accumulation of Treg following RT is the enhanced secretion of TGF‐β1 and TGF‐β2 by irradiated tumor cells [174], another potential mechanism may be that RT‐induced CCL2 expression in tumor cells promotes the CCR2‐dependent recruitment of TNFα‐producing monocytes and CCR2⁺ Treg cells, whose crosstalk via TNF‐α signaling leads to Treg activation and attenuates the antitumor efficacy of RT [175].Compared with nonirradiated tumors, TIL‐Treg from irradiated tumors exhibit similar or even greater suppressive capacity. Some studies also suggest that this suppression may be temporary [176]. Studies by Oweida et al. [177] and Ji et al. [178] employed in vivo models in which Treg were depleted using anti‐CD25 or anti‐CTLA‐4 antibodies. The results demonstrated a marked increase in tumor clearance, along with enhanced CD8⁺ T cell infiltration and function. These findings suggest that RT alone is insufficient to elicit durable tumor control or robust immune responses, whereas combining it with Treg ‐targeting strategies can significantly improve therapeutic efficacy.

Tumor‐associated MDSCs are a heterogeneous population of immature myeloid cells with immunosuppressive functions, primarily divided into monocytic MDSCs (M‐MDSCs) and polymorphonuclear MDSCs [179, 180]. Within the TME, MDSCs produce immunosuppressive cytokines such as IL‐10 and TGF‐β, deplete T cell function through PD‐L1 upregulation, and inhibit T cell activity and antitumor immunity via arginase‐1 (ARG‐1)‐mediated arginine depletion, inducible nitric oxide synthase (iNOS), and reactive oxygen species (ROS) release [179, 181, 182, 183]. Following RT, increasing number of TGF‐β and IL‐10 further promoting the recruitment and polarization of MDSCs and other immune cells. In turn, MDSCs can promote the production of Treg, creating a stronger feedback loop that sustains the immunosuppressive TME [184, 185]. This process may be driven by the upregulation of chemokines CCL2, CCL7, and CCL12 via STING activation, which facilitates MDSC accumulation in the TME through CCL2–CCR2 signaling [186]. In the study by Zhang et al. [187], activation of the CXCL1/CXCR2 and CCL2/CCR2 pathways appeared to specifically promote the recruitment of M‐MDSCs to the lungs, thereby suppressing T cell function and increasing the risk of pulmonary metastasis.

TAMs are another group of myeloid cells within the TME after RT and are considered the most abundant infiltrating cell type [188]. TAMs generally exhibit two polarization states: M1 (classically activated, tumoricidal) enhance antitumor immunity by secreting TNF‐α, IL‐1β, and IL‐6, and producing nitric oxide and ROS, directly killing tumor cells and inhibiting tumor growth [188]. M2 (alternatively activated, protumorigenic) macrophages are polarized by CSF‐1, IL‐4, IL‐10, IL‐13, and so on, leading to the secretion of IL‐10 and TGF‐β, the induction of Treg cells, and the promotion of tumor cell growth, migration, and metastasis, thus creating an immunosuppressive microenvironment [189]. Interestingly, the process of polarization is dynamic, with macrophages often displaying characteristics of both M1 and M2 types. Studies have shown that high‐dose irradiation (HDI) can increase the number of M2‐like TAMs in the tumor environment, potentially through inhibition of iNOS production. Notably, distinct radiation dosing regimens exert divergent regulatory effects on immunosuppressive cellular components within the TME. A preclinical study by Marine et al. [191] demonstrated that HDI (≥8 Gy per fraction) promotes the polarization of TAMs toward an immunosuppressive M2‐like phenotype, characterized by upregulation of ARG‐1, IL‐10, and TGF‐β. This shift impairs the cytotoxic function of CD8⁺ T cells and facilitates tumor progression [190, 191]. In contrast, moderate and low doses RT appear to suppress proinflammatory TNF‐α production and promote M1 polarization [167, 189, 192, 193, 194].

#### 3.3.4 Effects on Stromal Components

CAFs are a heterogeneous group of stromal cells within tumors, derived from normal fibroblasts but with distinct characteristics [195, 196]. CAFs often make up the majority of stromal cells in various cancers [197]. Previous research has shown that CAFs promote tumor progression, potentially by modulating the extracellular matrix and secreting immunosuppressive cytokines or growth factors (such as IL‐10 and TGF‐β), which support tumor proliferation, invasion, and metastasis [198, 199, 200, 201]. RT can recruit CAFs and appears to have varying effects on their functions [202]. However, growing evidence suggests that RT may alter certain CAFs, potentially limiting tumor growth and migration by forming a physical barrier against tumor progression [203]. In a study by Grinde et al. [204], both single HDI (1 × 18 Gy) and fractionated irradiation (3 × 6 Gy) eliminated the tumor‐promoting abilities of CAFs [26]. Nevertheless, due to CAF heterogeneity, their role in RT‐induced immune responses remains poorly understood [114].

### 3.4 Chemotherapy

**FIGURE 4:**
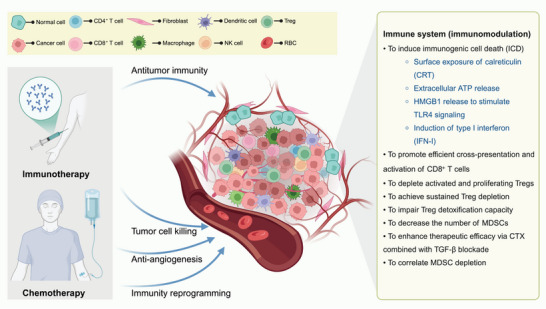
Immunomodulatory effects of chemotherapy. Chemotherapy can induce immunogenic cell death (ICD), characterized by calreticulin (CRT) exposure, extracellular ATP release, HMGB1 release, and type I interferon (IFN‐I) production, which collectively enhance dendritic cell activation and cross‐priming of CD8⁺ T cells. Certain agents (e.g., docetaxel, low‐dose cyclophosphamide) selectively deplete proliferating Treg cells, with sustained effects achievable through intermittent low‐dose regimens. Additionally, chemotherapy impairs Treg cell detoxification capacity, reduces myeloid‐derived suppressor cell (MDSC) levels, and synergizes with TGF‐β blockade to enhance antitumor immunity. These changes are associated with improved responses to neoadjuvant chemotherapy.

CT has been employed in cancer treatment since the 1940s [205], with its primary mechanisms involving cytotoxic effects such as DNA damage, synthesis inhibition, and microtubule disruption [206, 207]. Emerging evidence indicates that certain anticancer agents not only exert direct cytotoxic effects but also indirectly activate antitumor immune responses by releasing tumor antigens, attenuating tumor‐induced immunosuppressive networks, and enhancing tumor cell susceptibility to immune‐mediated attack [208, 209]. The Immunomodulatory effects of CT shows in Figure 4.

### 3.5 Immunostimulant Effects of CT

#### 3.5.1 Immunogenic Cell Death

CT not only directly eliminates tumor cells through cytotoxic effects but also potently activates host antitumor immunity by inducing ICD [210]. As we have systematically discussed the immunogenic mechanisms mediated by ICD in the preceding RT section, here we briefly summarize the key mediators of post‐CT immunomodulation. The immune‐activating effects of ICD primarily depend on the following DAMPs: (1) surface exposure of CRT, which serves as an “eat‐me” signal to promote DC phagocytosis and antigen uptake [211]; (2) active extracellular release of ATP, which activates the inflammasome via P2RX7 or P2RY2 receptors to induce IL‐1β secretion and immune cell recruitment [212, 213]; (3) HMGB1 release, which stimulates the TLR4 signaling pathway to enhance APC maturation [4.1]—studies show that concurrent administration of TLR4 agonists can augment ICD‐induced ICD and antitumor immune responses triggered by anthracyclines or oxaliplatin CT [214]; (4) IFN‐I production, which mimics viral infection signals through pathways such as TLR3 or cGAS–STING, thereby activating “viral mimicry” type immune responses [215, 216]. These DAMP signals act synergistically to significantly improve antigen cross‐presentation efficiency and induce potent CD8⁺ CTL responses.

Anthracyclines (e.g., doxorubicin, epirubicin) and other agents like oxaliplatin and cyclophosphamide (CTX) represent classic ICD inducers [217, 218]. Doxorubicin specifically promotes eIF2α phosphorylation, triggering ER stress and autophagy activation to enhance CRT and HMGB1 surface exposure, thereby boosting tumor immunogenicity [219, 220]. In contrast, while structurally similar to oxaliplatin, cisplatin lacks the essential DAMP‐release capacity required for ICD induction [220].

Preclinical studies by Sistigu et al. [216] demonstrated that anthracyclines activate TLR3 in tumor cells, driving IFN‐I and CXCL10 release. Notably, IFN‐I‐related gene signatures may serve as predictive biomarkers for anthracycline response in breast cancer patients [216]. Further supporting this, Wang et al. [221] revealed that oxaliplatin induces CRT exposure, ATP/HMGB1 release in laryngeal carcinoma cells, and activates DCs (upregulating CD83/CD86 and secreting IL‐6/TNF‐α), ultimately enhancing IFN‐γ production by CD8⁺ T cells to amplify antitumor immunity. While most DNA‐damaging chemotherapeutic agents can trigger apoptosis, they do not necessarily induce ICD. Studies by Martins et al. [222] demonstrated that cisplatin, etoposide, and mitomycin C fail to elicit ICD due to their inability to promote CRT translocation from the ER to the cell surface [222, 223]. Notably, the immunotherapeutic efficacy of ICD depends not only on tumor cell responsiveness but also on the host immune system's capacity to recognize DAMPs [224, 225]. For instance, loss‐of‐function polymorphisms in TLR4 or P2RX7 have been associated with poorer outcomes in breast and head and neck squamous cell carcinoma (HNSCC) patients receiving anthracycline‐based adjuvant CT [226, 227, 228]. However, this correlation was not observed in NSCLC, suggesting tumor type‐specific biological heterogeneity in ICD‐mediated immune responses [229]. In summary, ICD represents both a central mechanism underlying CT‐induced immune activation and a critical theoretical foundation for developing synergistic combinations of CT and IT.

#### 3.5.2 Antigen‐Presenting Cells

APCs are crucial bridges for activating T cells and directing tumor‐specific immune responses [230]. However, in most solid tumors, tumor‐infiltrating DCs often exhibit insufficient costimulatory molecule expression, maturation defects, or functional impairments that limit the priming and expansion of effector T cells [231]. Multiple chemotherapeutic drugs can directly induce DC maturation and enhance their immune‐activating functions. Studies have shown that microtubule‐destabilizing agents including vinca alkaloids can directly activate DCs, inducing the expression of IL‐1β, IL‐6, and IL‐12 while upregulating CD40, CD80, CD86, and MHC class II molecules, thereby enhancing their ability to activate T cells [232]. Subsequent research demonstrated that gemcitabine (Gem) could significantly restore the cross‐presentation capacity of tumor‐infiltrating DCs [233], reversing their semi‐mature state and enhancing tumor‐specific CD8⁺ T cell activation. This was further confirmed in an in vitro model by Nowak et al. [234], where the combination of Gem with an agonistic anti‐CD40 antibody achieved up to 80% cure rates in murine solid tumor models. However, while topoisomerase I inhibitors have been shown to promote DC maturation, under specific conditions they may also induce TH2 polarization and increase IL‐10 secretion, suggesting their effects on DCs may be regulated by the TME or combination treatment strategies [235]. Additionally, studies have shown that anthracyclines can indirectly activate DC maturation by inducing ICD and the release of DAMPs. In summary, chemotherapeutic drugs may play a key role in “resetting” the tumor immune microenvironment by directly or indirectly influencing the maturation state and antigen‐presenting capacity of DCs [130].

### 3.6 Immunosuppressant Effects of CT

#### 3.6.1 Off‐Target Effects

When chemotherapeutic agents are administered at or near the maximum‐tolerated dose (MTD), they frequently induce immunosuppression, primarily through potent cytotoxic and antiproliferative effects on various immune cell populations—most notably hematopoietic progenitors within the bone marrow. Among these adverse effects, CT‐induced neutropenia represents the most severe hematologic toxicity in cancer treatment [236, 237].

#### 3.6.2 Negative Regulation of Immune Suppressive Cells

Treg cells have been shown to be sensitive to CT drugs, particularly in activated and proliferating states [238]. In a study by Li et al. [239], Treg cells were significantly elevated in the peripheral blood of NSCLC patients compared with healthy individuals, and docetaxel was found to deplete activated Treg cells, thereby alleviating the immunosuppressive microenvironment [239]. Low‐dose CTX has been demonstrated in multiple studies to effectively deplete Treg cells [240, 241]. However, this depleting effect appears to follow a cyclical rhythm. In a study of breast cancer patients receiving daily CTX, the proportion of Treg cells significantly decreased during the first 42 days but rebounded to pretreatment levels by day 56 [242]. This finding suggests that under conditions of continuous CTX exposure, Treg cells may develop adaptive tolerance to drug‐induced depletion, implying that intermittent dosing strategies might be more effective than continuous administration for achieving sustained Treg cell depletion. The underlying mechanism may involve the intrinsically low ATP levels in Treg cells, which limit glutathione synthesis and impair their ability to detoxify CTX's active metabolite, phosphoramide mustard [243, 244]. Additionally, Treg cells lack the ABCB1 transporter, compromising their capacity to efflux toxic metabolites [245]. In a 4T1 model, Chen et al. [246] observed an over 80% tumor clearance rate when combining CTX with a TGF‐β neutralizing antibody, whereas TGF‐β blockade alone unexpectedly increased Treg cell numbers. Multiple studies have demonstrated that certain chemotherapeutic agents can enhance antitumor immune responses by suppressing MDSCs, thereby improving treatment outcomes. In a neoadjuvant study of breast cancer patients, regimens containing doxorubicin and CTX significantly reduced granulocytic MDSC (G‐MDSC) levels, with patients achieving pathological complete response (pCR) showing markedly lower posttreatment MDSC levels than non‐pCR patients, suggesting a correlation with therapeutic efficacy [247]. Another clinical trial enrolling mRCC and pancreatic cancer (PC) patients demonstrated that combining cytokine‐induced killer cell therapy with MDSC‐depleting CT significantly improved 1‐year survival rates and median OS [248]. Mechanistically, chemokine receptor CXCR2‐mediated MDSC migration was identified as a key regulatory target of CT. Highfill et al. [249] reported in a rhabdomyosarcoma model that CXCR2 blockade reduced MDSC tumor infiltration and synergistically enhanced anti‐PD‐1 efficacy. Low‐dose paclitaxel (PTX) was further shown to suppress p38 MAPK signaling in MDSCs, thereby inhibiting their proinflammatory cytokine secretion and function [250]. Notably, some agents like Gem and 5‐fluorouracil (5‐FU) may paradoxically activate the NLRP3 inflammasome in MDSCs, triggering IL‐1β secretion that promotes IL‐17 production by CD4⁺ T cells, ultimately suppressing antitumor immunity and diminishing CT efficacy [251]. Similarly, while CTX depletes Treg cells, a study by Ding et al. [252] revealed that CTX simultaneously expands immunosuppressive inflammatory monocytic myeloid cells (CD11b^+^Ly6C^hi^CCR2^hi^), which inhibit antitumor CD4⁺ T cell responses via the PD‐1/PD‐L1 axis and promote tumor relapse. These findings suggest that combining CTX with MDSC‐targeting agents (e.g., Gem) or other immunomodulatory strategies could optimize its therapeutic impact.

In most solid tumors, TAMs predominantly exhibit an M2‐polarized phenotype that strongly supports primary tumor growth and metastatic dissemination [253]. However, certain chemotherapeutic agents can reprogram these immunosuppressive TAMs toward an antitumoral M1 phenotype. A study by Carlos et al. [254] demonstrated that PTX, acting as a TLR4 agonist, effectively promotes the repolarization of TAMs from M2 to M1 phenotype, thereby enhancing antigen presentation and effector T cell recruitment. The underlying mechanism involves PTX's ability to “re‐educate” macrophages by inducing the expression of multiple proinflammatory genes, including TNF‐α, IL‐12, iNOS, and cyclooxygenase‐2, while simultaneously activating various immunomodulatory factors such as transcription factors, colony‐stimulating factors, adrenomedullin, and proinflammatory cytokines [61, 255]. These findings suggest that certain chemotherapeutic drugs may confer multiple immunostimulatory effects on the TME through M1 reprogramming of TAMs, potentially creating more favorable conditions for antitumor immunity [256].

## 4 Clinical Advances in Combined RT and/or CT with IT

In this section, we mainly analyze the potential synergy between RT/CT and IT, outline their roles within the tumor immune microenvironment, and summarize current clinical progress and key challenges in combination strategies, including dosing, fractionation, timing, and sequencing.

### 4.1 Clinical Progress of RT Combined with IT

RT can be categorized by dose size and fractionation into conventional fractionated RT (CFRT), macrofractionated RT, and hyperfractionated or single high‐dose RT [257]. While the immunostimulatory and immunosuppressive effects of RT have been discussed, the optimal dose and fractionation strategy for maximizing antitumor effects remain uncertain. Consequently, this area continues to be a key focus of both preclinical and clinical research.

#### 4.1.1 Dosing and Fractionation Strategy

Dose and fractionation are critical determinants of the immunomodulatory effects of RT. Research reported that a dose‐dependent rise in MHC‐I expression following single doses of 1, 4, 7, 10, or 25 Gy in human melanoma cells, with minimal expression at 1 Gy but over a twofold increase at 10–25 Gy within 48–72 h. This indicates that the response of MHC‐I to RT, both in vitro and in vivo, is dose dependent [116].

Low‐dose RT (LDRT) can enhance the immunogenicity of “cold” tumors, providing a basis for immunotherapies [258]. Studies show that LDRT can stimulate NK cells by upregulating effector proteins like IFN‐γ and TNF‐α through the p38 MAPK pathway, thereby enhancing NK cell cytotoxicity [259, 260]. This finding was reinforced by Cheda et al. [261] and Klug et al. [194]. Compared with effector T cells, LDRT (0.94 Gy) was found to induce significant apoptosis in Treg cells [262]. Notably, the application of low‐dose radiation has been shown to synergize with IT, enhancing antitumor efficacy [263]. In a phase I clinical trial [264], eight patients with immune‐cold tumors received LDRT combined with low‐dose CTX and ICIs showed substantial CD4+ T cell infiltration, dominated by Th1 characteristics, with reactive lesions observed in three patients, although no complete responses were noted.

In the meantime, single high‐dose local RT has been shown to modify the immuno‐inflammatory characteristics of tumors, doses higher than 20 Gy can induce the exonuclease TREX1, which degrades cytoplasmic DNA and inhibits STING‐mediated IFN‐I production in mice, thereby diminishing radiation‐induced immunogenicity [265, 266]. However, Hua Liang et al. [186] suggested that MDSCs may contribute to diminishing the antitumor effects of RT, and also indicated that HDI may promote MDSC recruitment. Together, these findings emphasize that radiation dose is a critical factor in optimizing the combination of RT and IT.

Fractionation shapes both the quality and magnitude of RT‐induced immune responses. Schaue and colleagues [267] found that compared with a single 15 Gy dose, fractionated treatment with a moderate dose of 7.5 Gy per fraction provided optimal tumor control and immune response while maintaining low levels of Treg cells. Although CFRT (1.8–2 Gy/day) is often used for larger tumor volumes (>5–10 cm^3^) and can minimize damage to surrounding healthy tissue [268]. The direct effects of RT on lymphatic organs and cells can negatively affect anticancer immune responses [30, 114, 269]. Stereotactic body RT (SBRT), a method that uses modern linear accelerators to deliver high doses of radiation to relatively small target lesions in fewer fractions [270, 271, 272], was demonstrated it can induce both local and distant immune effects by triggering the release of sufficient TAAs and ICD [273]. Recent studies in mouse models have demonstrated that an 8 Gy dose administered in three fractions not only provides comparable tumor control to single doses of 20 or 30 Gy, but also promoting distant antitumor effects [265, 274]. Due to its effective immune activation [275, 276], combining SBRT with IT may offer a promising new treatment approach [116]. In a study by Dewan et al. [274], only the combination of anti CTLA‐4 therapy and fractionated RT rather than single‐dose RT enhanced tumor response at the primary tumor site and induced distant effects at the secondary tumor site (*p* < 0.01). This combination approach has also been tested in clinical trials. Study demonstrated that SBRT induced greater CD8+ T cell proliferation and infiltration than low‐dose RT in CRC patients with microsatellite instability [277, 278, 279, 280].

Although studies have demonstrated that SBRT is involved in multiple immune‐activated antitumor processes [281], in the study by Kim et al. [282], the efficacy of combining nivolumab (anti‐PD‐1 monoclonal antibody) and ipilimumab (anti‐CTLA‐4 monoclonal antibody) in the treatment of Merkel cell carcinoma showed that the ORR for the IT‐only group and the combination SBRT group were 72 and 52%, respectively, with no significant difference between the two groups (*p* = 0.26). This indicates that the addition of SBRT did not significantly improve the efficacy. Similarly, there is evidence that high‐dose RT can lead to a significant increase in TGF‐β [281], which is generally believed to promote tumor progression [201, 283]. As a result, relying on SBRT alone may not be sufficient to effectively initiate an antitumor immune response [3]. In a phase II clinical trial (NCT02239900) evaluating the efficacy of ipilimumab in combination with SBRT for metastatic lesions in the liver or lungs, an interesting finding was observed: tumors exposed to low‐dose irradiation were more likely to respond than lesions located further away (31 vs. 5%, *p* = 0.0091) [284]. This suggests that combining SBRT with low‐dose RT may represent a promising new approach for combined IT.

In summary, these findings highlight that radiation dose and fractionation are crucial factors in developing a successful combination of RT and IT. A deeper understanding of the immune mechanisms underlying RT may lead to more efficient and effective use of RT, while also guiding the design of immuno‐adaptive RT studies, thus opening new possibilities for cancer treatment [285].

#### 4.1.2 Time and Sequencing

Herrera et al. [264] observed changes happened in the TME ten days after RT. Through genetic profiling, they found that a large influx of lymphocytes, NK cells, macrophages, and DCs occurred on the fifth day following RT, while cell conditions varied every day. This observation suggests that immune responses after RT are time dependent, indicating that the effectiveness of combined RT and IT could be significantly influenced by the timing of treatment. Therefore, administering IT at different time points—before, during, or after RT—may yield different clinical outcomes.

Modern IT employs various treatment mechanisms, with immune checkpoint blockade (ICB) being the most widely utilized approach. ICB works by removing inhibitory signals that prevent T cell activation, thereby enabling tumor‐reactive T cells to generate an antitumor response [286]. Due to the significant clinical efficacy of PD‐1 and CTLA‐4 inhibitors, as well as their broad applicability across various tumor types [287], this review will primarily focus on discussing these two classes of drugs.

**Evidence from preclinical studies provides a foundation**.Study conducted by Ban et al. [288] demonstrated that in immunocompetent mouse model of NSCLC that combining anti‐PD‐1 therapy (1 day prior to RT) with RT (4 Gy for three fraction) significantly improved therapeutic outcomes compared with either PD‐1 inhibition or RT alone. In a study by Verbrugge et al. [289], the mouse model with triple‐negative breast cancer (TNBC) was given the simultaneous use of single or low‐dose fractionated RT combined with PD‐1 blockade, which significantly enhanced antitumor responses through activating CD8+ T cell. Above effects were attributed to the rapid upregulation of PD‐1 and PD‐L1 following RT [290]. Similarly, Dovedi et al. [26, 291] concluded that the timing of anti‐PD‐L1 antibody administration is critical for maximizing therapeutic outcomes. They found that the best results were achieved when PD‐L1 blockade was administered either simultaneously with or immediately following RT. Delaying PD‐L1 infusion for 1 week eliminated the synergistic effect between RT and PD‐1 inhibition [26, 291]. In contrast, preclinical studies that using anti‐CTLA‐4 therapy suggest a different timing sequencing [274]. Young et al. [292] investigated the use of anti‐CTLA‐4 therapy as a neoadjuvant treatment in mice receiving 20 Gy tumor RT. When administered 7 days before RT, anti‐CTLA‐4 led to complete tumor regression and 100% survival. However, when administered 1 or 5 days after RT, only 50% of tumor‐bearing mice showed complete tumor resolution, with median survival times of 92 and 53 days, respectively. This highlights that administering anti‐CTLA‐4 prior to RT may effectively trigger immune responses.

However, research from Wei et al. [293] found that administering anti‐PD‐1 therapy 1 day after 8 Gy RT in CRC mice model triggered robust anticancer immunity and systemic effects. In contrast, initiating IT before RT significantly reduced the treatment's efficacy. Similarly, Ban et al. [288] found that IT given before RT did not show enhanced efficacy, possibly due to differences in RT doses, fractionation regimens, and IT protocols across studies, which complicates direct comparison.

In summary, in the preclinical studies reviewed above, the current data on anti‐CTLA‐4 treatment remain insufficient to provide clear recommendations regarding optimal sequencing. However, existing preclinical evidence for anti‐PD‐1/PD‐L1 treatments strongly supports simultaneous or sequential using with RT. These studies demonstrate various timing strategies for combining RT and ICIs, including before, concurrent, and after ICIs with RT. While all these regimens have shown promising antitumor effects, the comparability of these results is limited due to the lack of systematic research on optimal timing. Thus, while current studies show the effectiveness of combining RT with IT, they have not clearly defined the optimal timing, further research is needed to systematically assess.

Findings from clinical research highlight the real‐world impact and feasibility. Theelen et al. [294] conducted a pooled analysis of two randomized trials on metastatic NSCLC and found that the combination of pembrolizumab and RT was superior to RT alone in terms of response rate (NCT02492568, NCT02444741). This suggests that RT may enhance the efficacy of IT.

The PACIFIC trial (NCT02125461) is a double‐blind phase III landmark randomized study that evaluated the effectiveness of durvalumab as consolidation therapy following RT in patients with locally advanced unresectable NSCLC [295]. The results demonstrated a significant improvement in median progression‐free survival (PFS), with the durvalumab group showing 16.8 months compared with 5.6 months in the control group [296]. The ORR was also higher with 28.4 versus 16.0%, respectively. These findings highlight the potential of timely IT after RT to improve patient outcomes [297]. Similarly, the ETOP NICOLAS trial (NCT02434081), conducted by Peters et al. [298], demonstrated that the addition of nivolumab to RT was safe and enhanced overall treatment efficacy, despite some adverse events such as anemia (47.5%) and pneumonia (42.5%) existed. The improved efficacy observed in this trial may be attributed to the immunosuppressive effects of RT, which subsequently lead to increased PD‐1/PD‐L1 expression, enhancing the immune response [292].

For anti‐CTLA‐4‐based treatment, clinical trial data may support sequential therapy over simultaneous IO‐RT could offer more effective outcomes in specific patient populations. For instance, in patients with advanced tumors(lung metastasis), combining ipilimumab with synchronous SBRT (four fractions with a total dose of 50 Gy or 10 fractions with a total dose of 60 Gy) showed a clinical benefit rate of 26%. However, for patients who received sequential RT and lung lesion irradiation, the clinical benefit rate increased to 42% [284]. Conversely, the JAVELIN HEAD AND NECK100 study by Cohen et al. [299] failed to show similar improvements in efficacy. This study compared the combination of avelumab (administered on days 8, 25, and 39 of concurrent chemoradiotherapy) with avelumab maintenance therapy and standard chemoradiotherapy. However, the study did not meet its primary endpoint, as there was no significant difference in PFS or OS between the two groups. The reasons for these negative findings remain unclear and wait further investigation.

The optimal sequencing—whether presequence, sequential, or concurrent—has yet to be formally evaluated and compared in large‐scale clinical trials. In recent years, an increasing number of clinical trials have focused on this issue.

A randomized phase 1b SABRseq trial (NCT03307759), published in 2023, investigated the optimal order of stereotactic ablation RT (SABR) and anti‐PD‐1 checkpoint blockade that performing SABR either before or after pembrolizumab treatment led to favorable PFS and OS, with no significant differences in safety [300]. These promising results suggest that further evaluation in larger randomized trials is needed. Additionally, the ongoing ECOG‐ACRIN (EA5181) phase III randomized trial (NCT04092283) [301] is evaluating the efficacy endpoints of SBRT followed by or concurrent with pembrolizumab in patients with metastatic NSCLC, with subjects currently being recruited. As these studies progress, they are expected to provide valuable insights and evidence on the timing of radio‐IT combinations, which will be crucial for optimizing clinical treatment strategies.

Notably, the combination of RT with different ICIs, such as anti‐PD‐1 and anti‐CTLA‐4 antibodies, has yielded heterogeneous clinical outcomes. These discrepancies may stem from the distinct mechanisms by which each ICI modulates the immune response. Anti‐CTLA‐4 primarily acts at the priming phase of T cell activation within lymphoid tissues, enhancing the breadth of the antitumor T cell repertoire, whereas anti‐PD‐1 predominantly restores effector T cell function in the TME. Consequently, their interactions with RT—which itself influences both antigen release and immune cell recruitment—may differ depending on the immunological context of the tumor. Furthermore, RT‐induced upregulation of PD‐L1 may render anti‐PD‐1 therapy more effective in some settings, while the ICD triggered by radiation could synergize better with the priming‐enhancing effects of anti‐CTLA‐4 in others. These mechanistic nuances highlight the need for rational trial design that considers ICI mechanism of action, timing relative to radiation, and tumor‐intrinsic immune contexture. Future studies should incorporate immunophenotyping and spatial profiling to guide optimal combinations tailored to individual tumor immune landscapes. Representative ongoing or completed clinical trials investigating the combination of ICIs with RT are summarized in Table 1.

**TABLE 1:** Clinical trials combining radiotherapy with immunotherapy.

Study	Year	Disease	Radiotherapy	ICIs	Schedule	Result	References
**Lung cancer**							
NCT02221739	2015	Metastatic NSCLC	Focal radiation therapy (6 Gy × 5 or 9 Gy × 3)	Ipilimumab	ICI concomitant RT	Objective response in 18% (seven out of 39), disease control in 31% (12 out of 39); median OS 7.4	[302]
NCT02463994	2015	Metastatic NSCLC	Hypofractionated image‐guided radiotherapy (HIGRT)	MPDL3280A (PD‐L1 antibody)	ICI concomitant RT	Completed study evaluating the synergistic effect of HIGRT combined with PD‐L1 blockade (MPDL3280A), with overall response rate assessed by mRECIST 1.1 as the primary endpoint	/
NCT03102242	2020	Unresectable stage III NSCLC	Focal radiation therapy (6 Gy × 5 or 9 Gy × 3)	Atezolizumab	ICI before RT + continued after RT	Disease control rate at 12 weeks 74.2%, median PFS 30.0, 24‐month OS rate 73.7%; 27.4% experienced grade 3+ irAEs.	[303]
NCT03509584	2018	NSCLC	Part 1a: Nivolumab with hypofractionated RT (24 Gy in 3 fractions) (bone metastasis) Part 1b: Nivolumab and ipilimumab with hypofractionated RT (24 Gy in 3 fractions)(bone metastase) Part 2a: Nivolumab with hypofractionated RT (24 Gy in 3 fractions) (outside the brain) Part 2b: Nivolumab and ipilimumab with hypofractionated RT (24 Gy in 3 fractions) (outside the brain)	Nivolumab alone or nivolumab + ipilimumab	ICI concomitant RT	Study focused on evaluating the safety of hypo‐fractionated radiotherapy combined with nivolumab, alone or with ipilimumab; safety assessed primarily by incidence of immune‐related adverse events over 48 weeks	/
NCT03825510	2022	Metastatic NSCLC	Nivolumab or pembrolizumab with SBRT	PD‐1 blockade immunotherapy agent	Unclear timing	Assessing impact of SBRT plus immunotherapy on PFS and OS vs. immunotherapy alone; includes abscopal effect and toxicity evaluation	/
NCT03237377	2021	Stage III resectable NSCLC	Arm 1: 3 cycles × durvalumab with RT (1.8–2.0 Gy per fraction, daily ×5/week) Arm 2: 3 cycles x durvalumab and tremelimumab with RT (5 days per week in once daily fractionation, 1.8–2.0 Gy per fraction)	Durvalumab ± tremelimumab	ICI concomitant RT	Toxicity (CTCAE v4.0), feasibility of neoadjuvant RT + durvalumab ± tremelimumab, surgical complications/mortality, pathologic and radiologic response rates, RFS, OS	/
NCT03801902	2021	Stage II–III unresectable or inoperable LA‐NSCLC with PD‐L1 >50%	Arm I: 13 cycles × durvalumab with ACRT (60 Gy in 15 fractions) Arm II: 13 cycles × durvalumab with standard RT (60 Gy in 30 fractions)	Durvalumab	ICI concomitant RT	No dose‐limiting toxicities (DLTs) in cohort 1; 1 unrelated DLT in cohort 2. 85% in ACRT and 75% in CONV received second dose of durvalumab in first 8 weeks. Grade 3–5 adverse events reported but mostly unrelated to therapy; immunoradiotherapy with durvalumab appears safe and feasible	[304]
**Breast cancer**							
NCT03807765	2020	Breast cancer brain metastases	Stereotactic radiosurgery (SRS) 15–24 Gy	Nivolumab	ICI before RT	Median intracranial control 6.2 months (95% CI, 3–14 months), 6‐month control rate 55%, 12‐month control rate 22%; no dose limiting toxicities	[305]
NCT02563925	2022	Breast cancer brain metastases (BCBM), HER2− and HER2+ cohorts	Whole brain RT (30 Gy/10 fractions) or SRS	Tremelimumab (anti‐CTLA‐4) ± trastuzumab (HER2+ cohort)	Peri‐RT (concurrent or near RT)	At 12 weeks, extracranial disease control rate (DCR) was 10% for HER2‐negative and 33% for HER2‐positive; no grade 4 adverse events linked to therapy; limited efficacy in HER2‐, promising outcomes in HER2+	[306]
NCT03366844	2020	HR+/HER2− metastatic breast cancer	RT (5 × 4 Gy)	Pembrolizumab (anti‐PD‐1)	ICI before RT	No objective responses in eight patients; median PFS and OS were 1.4 and 2.9 months, respectively; 87.5% experienced adverse events (AEs), including one grade 3 aspartate aminotransferase (AST) elevation; study terminated early	[307]
NCT02730130	2019	Metastatic triple‐negative breast cancer (TNBC)	3000 cGy in 5 fractions	Pembrolizumab (anti‐PD‐1)	ICI concomitant RT	ORR 17.6% (three out of 17), including three complete responses (CRs); median PFS not reported; 4 grade 3 AEs (fatigue, lymphopenia, infection); no grade 4 AEs or treatment‐related deaths	[308]
NCT02499367	2019	Metastatic triple‐negative breast cancer (TNBC)	3 × 8 Gy	Nivolumab (anti‐PD‐1)	ICI after RT	ORR 20% (13/66), CR 3%, PR 17%; best responses in doxorubicin (ORR 35%) and cisplatin (ORR 23%) cohorts; higher TILs and PD‐L1 expression correlated with response	[309]
NCT03430479	2022	HER2‐negative MBC	8 Gy single fraction (bone)	Nivolumab	ICI concomitant RT	Cohort A: ORR 11% (90% CI 4–29%), DCR 39% (90% CI 23–58%), mPFS 4.1 months; Cohort B: ORR 0%, DCR 0%, mPFS 2.0 months; 1 grade 3 AE in cohort B	[310]
NCT03004183	2022	mTNBC	SBRT	Pembrolizumab	ICI after RT	Clinical benefit rate (CBR) was 21.4% (CR 7.1%, PR, stable disease 10.7%); responders had median duration of treatment (DoT) of 9.6 months and median overall survival (OS) of 14.7 months; median OS for all patients was 6.6 months; responders showed a marked increase in CD8+ T cells.	[311]
NCT03464942	2022	Advanced TNBC	SABR (20 Gy/1 fraction or 24 Gy/3 fraction)	Atezolizumab	ICI after RT	Median PFS: 2.5‐3.1 months; 12‐month OS: 78% (20 Gy), 57% (24 Gy); ORR 8% (PD‐L1+), 7% (PD‐L1−)	[312]
NCT03036488	2023	TNBC	Adjuvant RT given per protocol; timing analyzed as concurrent or sequential with adjuvant pembrolizumab/placebo	Pembrolizumab	ICI concomitant RT	EFS benefit with pembro vs. placebo regardless of RT use; grade 3–5 treatment‐related adverse events occurred in 5.9% (pembro) vs. 2.7% (placebo) with RT, and 7.5 vs. 2.9% without RT; immune‐related adverse events were comparable. The combination of pembrolizumab and RT was tolerated.	[313]
**HNSCC**							
NCT02952586	2021	Locally advanced HNSCC	IMRT, 70 Gy (35 fractions) over 7 weeks, with cisplatin 100 mg/m^2^ Q3W	Avelumab	Avelumab + chemo‐RT vs. placebo + chemo‐RT	No PFS/OS benefit; median PFS NR (avelumab) vs. NR (placebo), HR 1.21 (95% CI 0.93–1.57); higher serious AEs in avelumab group	[314]
NCT02999087	2021	LA‐SCCHN (locally advanced squamous cell carcinoma of head and neck)	RT: 70 Gy/6.5 wks IMRT + weekly cetuximab	Avelumab	Avelumab 10 mg/kg Q2W during RT + maintenance ×12 months	Cisplatin‐unfit: 2‐year PFS 44 vs. 31% (HR 0.85); distant metastasis reduced (HR 0.31); OS similar; cisplatin‐fit: futility boundary crossed, standard‐of‐care favored (HR 1.27)	[315]
NCT03040999	2022	LA‐HNSCC (locally advanced head and neck squamous cell carcinoma)	CRT 70 Gy/35 fractions + cisplatin 100 mg/m^2^ Q3W	Pembrolizumab	ICI before, concomitant, and after RT	EFS HR 0.83 but not statistically significant; grade ≥3 AEs: 92.2 vs. 88.4%;	[316]
NCT03452137	2018	Locally advanced SCCHN	Definitive local/regional therapy (surgery and/or chemoradiotherapy) completed prior to randomization	Atezolizumab	ICI after RT	Study ongoing	[317]
NCT03765918	2019	Resectable locally advanced HNSCC	SOC (radiotherapy ± cisplatin) postsurgery	Pembrolizumab	ICI before surgery + ICI after surgery	Ongoing; dual primary endpoints: major pathological response (≤10% invasive tumor) and event‐free survival; recruitment ongoing, results pending	[318]
NCT03700905	2020	Locally advanced HNSCC	SOC adjuvant chemoradiotherapy	Nivolumab ± ipilimumab	ICI before RT	Ongoing; evaluating DFS benefit of neoadjuvant nivolumab ± ipilimumab vs. standard adjuvant CRT	[319]
NCT02608385	2018	Metastatic solid tumors	Multisite SBRT (2–4 metastases, 30–50 Gy in 3–5 fractions)	Pembrolizumab	ICI after RT	ORR 13.2%; mPFS 3.1 mo; mOS 9.6 mo; IFN‐γ gene upregulation linked to abscopal response	[320]
NCT02684253	2018	Metastatic HNSCC	SBRT (9 Gy × 3 to 1 lesion)	Nivolumab	ICI concomitant RT	No ORR (34.5 vs. 29.0%, *p* = 0.86) or survival benefit with SBRT; similar safety	[321]
**Cervical cancer**							
NCT05173272	2023	Locally advanced cervical cancer (FIGO 2018 Ib3‐IIIc2)	Concurrent chemoradiotherapy	Slulimumab	ICI before RT	Recruiting; Primary outcome: progression‐free survival (PFS) improvement	/
NCT02635360	2017	Locally advanced cervical cancer (LACC)	Concurrent chemoradiotherapy (CRT)	Pembrolizumab	Arm 1: ICI after RT Arm 2: ICI concomitant RT	Similar CR and PR rates at 3‐month PET/CT in both arms; fewer PD cases in Arm 2	[322]
NCT03833479	2022	High‐risk locally advanced cervical cancer (partial/complete response after CRT)	Definitive concurrent chemoradiation	Dostarlimab	ICI after RT	Primary endpoint: progression‐free survival; results not yet available	[323]
NCT05492123	2024	Locally advanced cervical cancer	Cisplatin‐based chemoradiation	Nivolumab + ipilimumab induction, then concurrent nivolumab with chemoradiation	ICI before and concomitant RT	Primary: 3‐year progression‐free survival	/
NCT03612791	2024	Locally advanced cervical cancer	Standard concurrent chemoradiotherapy (CRT)	Atezolizumab	ICI concomitant RT + ICI after RT	Primary: progression‐free survival Not reported	/
NCT05311566	2022	Early to locally advanced cervical cancer (FIGO IB2‐IIIB)	Concurrent radiotherapy combined with cisplatin CT for 6–8 weeks	Camrelizumab	ICI concomitant RT	Recruiting, safety, and efficacy outcomes pending	/
NCT04221945	2025	Locally advanced cervical cancer	Concurrent chemoradiotherapy (standard CRT)	Pembrolizumab	ICI concomitant RT	Ongoing, primary endpoints: PFS and OS pending	/
**Melanoma**							
NCT01497808	2015	Melanoma	SBRT Lung/bone: 8 Gy × 2 (DL1), 8 Gy × 3 (DL2) Liver/Subcutaneous: 6 Gy × 2 (DL1), 6 Gy × 3 (DL2)	Ipilimumab	ICI after RT	In patients, RT plus anti‐CTLA‐4 induced tumor regression, but resistance occurred due to PD‐L1 upregulation; adding anti‐PD‐L1 overcame this resistance and led to durable responses.	[324]
NCT02239900	2017	NSCLC, CRC, RCC, others	50 Gy in 4 fractions; 60 Gy in 10 fractions	Ipilimumab	ICI after RT	Ipilimumab combined with SABR was generally well tolerated.	[325]
NCT02608385	2018	Ovarian, endometrial, CRC, others	30–50 Gy × 3–5 fractions	Pembrolizumab	ICI after RT	ORR 13.2%; median OS 9.6 mo; PFS 3.1 mo; well tolerated (DLT in 6 pts)	[320]
NCT02303990	2018	Cohort 1: NSCLC, melanoma (progression on anti‐PD‐1) and Cohort 2: pancreas, breast, H&N, colon, kidney (no prior anti‐PD‐1)	High‐frequency radiotherapy (HFRT): 8 Gy × 3 fractions for first half; 17 Gy × 1 fraction for second half	Pembrolizumab	ICI before RT	20/24 patients (83%) had grade 1–2 treatment‐related AEs; Stratum 1 (*n* = 12): 2 prolonged responses (9.2, 28.1 months); Stratum 2 (*n* = 12): 1 complete response, 2 prolonged SD (7.4, 7.0 months).	[326]

/: no corresponding publication available; see www.clinicaltrials.gov for details.

### 4.2 Clinical Progress of CT Combined with IT

As discussed in the preceding section, CT exerts immunomodulatory effects beyond its direct cytotoxicity against tumor cells. These additional mechanisms provide a strong rationale for the combination of CT and IT, which may hold substantial therapeutic potential.

#### 4.2.1 Combination of CT and ICIs

Notably, certain chemotherapeutic agents have also been shown to upregulate the expression of immune checkpoint molecules. Platinum‐based compounds not only promote antigen presentation and induce ICD, but also enhance PD‐L1 expression across various tumor types. For instance, in studies by Park et al. [327], both cisplatin and oxaliplatin were found to upregulate PD‐L1 expression in HNSCC, a finding that was similarly observed in NSCLC [328, 329]. In ovarian cancer, cisplatin was shown to simultaneously induce antigen presentation, ICD, and a marked increase in PD‐L1 expression [330]. In addition, a range of other chemotherapeutic agents—including doxorubicin, PTX, topotecan, and Gem—have been reported to induce PD‐L1 expression in breast cancer and pancreatic ductal adenocarcinoma, occasionally accompanied by the upregulation of CTLA‐4 [331]. These findings suggest that CT‐induced PD‐L1 expression may serve as a predictive biomarker, already utilized for treatment stratification in NSCLC and providing a compelling rationale for integrating CT with ICIs [332].

In patients with metastatic nonsquamous NSCLC, platinum‐based CT remains the standard first‐line treatment [333]. Pembrolizumab, an anti‐PD‐1 antibody, has become the preferred option in individuals with high PD‐L1 expression; however, the clinical benefits of its combination with CT continue to be evaluated. The phase III KEYNOTE‐189 [334] trial assessed the efficacy of pembrolizumab in combination with pemetrexed and platinum‐based CT in treatment‐naïve patients with metastatic nonsquamous NSCLC lacking EGFR or ALK alterations. The combination group demonstrated a median PFS of 8.8 months, compared with 4.9 months in the control group (HR = 0.52). The 12‐month OS rate was 69.2% in the pembrolizumab arm versus 49.4% in the placebo arm (HR = 0.49). Importantly, survival benefits were observed across all PD‐L1 expression subgroups. The incidence of grade ≥3 adverse events was comparable between groups (67.2 vs. 65.8%), indicating that the regimen significantly improves prognosis with manageable toxicity. Similarly, the phase III KEYNOTE‐407 RCT reported consistent results in patients with metastatic squamous NSCLC. The combination of pembrolizumab with carboplatin plus PTX or nab‐PTXl significantly prolonged median PFS compared with CT alone [335].

Phase III RCTs such as IMpower133 [336], CASPIAN [337], and ASTRUM‐005 [338] have demonstrated that the addition of ICIs to platinum‐etoposide CT significantly prolongs both PFS and OS in patients with extensive‐stage small cell lung cancer (ES‐SCLC), with an acceptable safety profile. As a result, both the US FDA and the European Medicines Agency have approved atezolizumab or durvalumab in combination with platinum‐based CT plus etoposide as the first‐line standard of care for ES‐SCLC [339]. Nevertheless, the combination of CT and IT has yielded mixed outcomes in other tumor types. In the KEYNOTE‐048 trial [340], previously untreated patients with recurrent or metastatic HNSCC received pembrolizumab either as monotherapy, in combination with platinum plus 5‐FU, or were treated with cetuximab plus platinum and 5‐FU. Pembrolizumab monotherapy significantly improved OS in patients with a PD‐L1 combined positive score ≥1 or ≥20 and was noninferior to the cetuximab‐based regimen in the overall population. The combination of pembrolizumab with CT resulted in a statistically significant OS benefit across all patients. However, neither pembrolizumab‐based regimen improved PFS, and both combination arms were associated with a high incidence of grade ≥3 adverse events (85 and 83%, respectively). IMpassion130 was the first phase III trial [341] to evaluate the clinical utility of ICIs in combination with standard CT in the first‐line treatment of TNBC. In this trial, patients with previously untreated unresectable or metastatic TNBC were randomized to receive atezolizumab plus nab‐PTX (A+nP) or placebo plus nab‐PTX. Among patients with PD‐L1‐positive tumors, the combination therapy significantly prolonged both PFS (7.5 vs. 5.0 months) and OS (approximately 25.0 vs. 15.5–17.9 months) [342]. However, in the intention‐to‐treat population, the improvement in OS did not reach statistical significance. Moreover, the atezolizumab group experienced a higher incidence of treatment‐related adverse events and drug discontinuation compared with the placebo arm.

#### 4.2.2 Dosing and Time Sequencing

Notably, the immunomodulatory effects of CT are not solely determined by the class of agents used, but are also profoundly influenced by factors such as dosage, scheduling, and the temporal sequence of combination with IT, all of which shape distinct immune microenvironmental landscapes and ultimately govern the synergistic potential of chemo‐IT [343].

The sensitivity of immune cells to CT depends on both drug dosage and treatment timing. In a multiple myeloma model, low‐dose CTX administered every 45 days blocked Treg cell regeneration and restored antitumor immunity, leading to prolonged survival and prevention of relapse [344, 345, 346]. In contrast, more frequent dosing every 7 or 21 days failed to improve therapeutic outcomes and even accelerated tumor progression [347]. Chang et al. [348] demonstrated that compared with conventional MTD regimens, a dose‐dense CT protocol (cisplatin+PTX) enhanced macrophage recruitment and tumor‐specific CD8⁺ T cell responses in a murine ovarian cancer model. These findings were preliminarily validated in a clinical cohort of 14 patients [348]. Furthermore, studies have shown that noncytotoxic low doses of doxorubicin, PTX, methotrexate (MTX), and mitomycin C can directly improve DC‐mediated antigen presentation to T cells. This effect correlates with upregulated expression of CD80, CD86, CD40, and MHC class II molecules on DCs, along with IL‐12p70‐dependent secretion [349, 350].

In summary, CT exhibits dose‐ and TME‐dependent immunomodulatory properties. Optimizing drug selection and administration schedules represents a critical strategy for maximizing its immune‐regulating potential.

Liu et al. [351] demonstrated that low‐dose MTX downregulates IL‐2 expression while upregulating Foxp3 in T cells in vitro, an effect similarly observed in vivo. In contrast, high‐dose MTX failed to elicit such immunoregulatory responses, suggesting a dose‐dependent immunomodulatory role for low‐dose MTX [343]. Likewise, CTX administered at low doses (50–100 mg/kg intraperitoneally in mice; 200–300 mg/m^2^ intravenously in humans) has been shown to selectively deplete Treg cells, thereby enhancing antigen‐specific T cell responses [343]. This finding is supported by Banissi et al. [352], and may help explain why CT regimens at doses far below standard cytotoxic thresholds are strategically employed to augment tumor vaccine efficacy, rather than for direct tumoricidal purposes. In recent years, low‐dose CTX has been investigated across multiple clinical trials for its immunomodulatory potential. For instance, Laheru and colleagues [353] evaluated the use of GM‐CSF alone or in sequential combination with CTX (250 mg/m^2^) in patients with advanced PC. The combination group demonstrated enhanced CD8⁺ T cell responses and prolonged median OS (4.3 vs. 2.3 months in controls). In a phase II trial, Walter et al. [354] reported that administering a single low dose of CTX (300 mg/m^2^) prior to IT significantly reduced Treg cell levels, which correlated with improved immune responses and longer OS. Specifically, patients with reduced Treg cell levels who received IMA901 exhibited a median OS of 33.1 months. Notably, high‐dose CT does not appear to confer additional immunological benefit beyond its cytotoxic effects. Banissi et al. [352] further showed that high‐dose temozolomide (10 mg/kg daily for 21 days or 30 mg/kg for 5 days) did not significantly alter the Treg/CD4⁺ cell ratio, in contrast to the more pronounced immunomodulatory impact observed with low‐dose regimens. Mechanistically, low‐dose CT may activate host danger recognition pathways and tissue repair programs, leading to the release of immunostimulatory cytokines and chemokines. These signals in turn promote DC maturation, enhance antitumor T cell expansion, and increase infiltration of cytotoxic immune cells into the TME [343, 355]. Conversely, high‐dose CT exerts broad toxicity across multiple lymphocyte subsets, lacking immunological selectivity [356]. While high‐dose CT remains under investigation in combination with cancer vaccines or adoptive cell therapies, the optimal dosing strategy remains unresolved [357]. Current evidence suggests that the immune perturbations induced by CT are highly dose dependent and patient specific [358]. Therefore, clinical application of chemo‐IT combinations must be approached with caution, and further prospective studies are urgently needed to elucidate optimal regimens [359].

Beyond dosage, the scheduling and timing of CT administration profoundly influence its immunomodulatory effects. Preclinical studies have demonstrated that rhythmic dosing of agents such as CTX, PTX, or temozolomide can suppress Treg cell activity in murine models [352, 360, 361]. In clinical settings, Nars and Kaneno [362] reported that metronomic CTX not only significantly reduced Treg cell frequencies but also mitigated the functional suppression of T and NK cells. Similarly, in an HPV tumor‐bearing mouse model developed by Peng et al., a single prevaccination dose of CTX achieved comparable immune activation to prolonged daily low‐dose administration, while effectively avoiding depletion of CD8⁺ T cells [367]. Notably, an intermittent 6‐day cyclic regimen of the same CT agent induced more durable immune activation and prolonged tumor regression. However, extending this to two successive 6‐day cycles of CTX paradoxically led to increased infiltration of Foxp3⁺ Treg cells and reduced expression of cytolytic effector molecule perforin within the TME—suggesting a rebound in immunosuppressive dominance [365, 366]. These findings underscore the immunological sensitivity to CT timing and highlight that “rhythmic” or continuous dosing does not inherently translate to optimal immune synergy. Instead, the immunostimulatory versus immunosuppressive balance is dynamic and context dependent. Thus, tailoring the sequence and intervals of chemo‐IT regimens in specific tumor and drug contexts is critical for maximizing synergistic efficacy [363, 364].

In a preclinical study by Salem et al. [359], a single low‐dose administration of CTX (4 mg) in mice led to a marked expansion of peripheral immature DCs between days 8 and 16 posttreatment. When a TLR3 agonist, poly(I:C), was administered during this peak of DC expansion, it significantly enhanced DC activation and boosted the efficacy of adoptive T cell therapy. These findings suggest that the recovery phase following early lymphodepletion (days 1–4), particularly between days 5 and 18, represents a critical window of heightened immunostimulatory potential. This window may be leveraged to occupy immunological “niches” previously dominated by tolerogenic or tumor‐promoting immune cells, thereby synergizing with CT‐induced cytokine and chemokine fluxes to enhance antitumor responses [368, 369]. In many models, a “chemotherapy priming” strategy has emerged as the predominant paradigm. For example, in the 3Cl‐8 Friend leukemia mouse model, Moschella et al. [368] demonstrated that adoptive IT administered 5 h to 1 day after a single CTX dose (83 mg/kg) produced the most robust antitumor effects, whereas delaying treatment to day 3 abolished efficacy. This optimal timing coincided with CTX‐induced expansion of Th1, Th17, and activated CD25⁺CD4⁺Foxp3^−^ effector T cells, along with modest suppression of Treg cell populations. Other preclinical studies have yielded comparable findings, further supporting the importance of temporally optimizing IT delivery relative to CT‐induced immune dynamics [370]. In a clinical study conducted by Palermo et al. [371], 36 patients with stage II–IV melanoma received dacarbazine 1 day prior to vaccination. Transcriptomic analyses revealed upregulation of genes associated with immune responses and leukocyte activation. Moreover, there was a significant increase in Melan‐A‐specific, tumor‐reactive, and durable effector memory CD8⁺ T cells, with a more diverse TCR repertoire compared with vaccination alone. Interestingly, prior IT may also sensitize patients to subsequent CT. In a study by Antonia et al. [372], 29 patients with ES‐SCLC were repeatedly vaccinated with DCs transduced with full‐length wild‐type p53. Although the vaccine alone elicited a clinical response in only one patient, CT administered immediately after vaccination resulted in a remarkably high ORR of 61.9%. Similarly, findings by Wheeler et al. [373] demonstrated a logarithmic correlation between the magnitude of vaccine‐induced immune activation and the clinical benefit observed during subsequent CT.

Collectively, these studies suggest that the synergistic efficacy between CT and IT is not governed by a single factor but instead arises from the complex interplay of CT dose, drug class, timing of immunologic intervention, and the patient's baseline immune landscape. Moving forward, systematic “immune profiling” will be essential to elucidate the comprehensive immunologic impact of various chemotherapeutic regimens and to guide the development of rational, personalized treatment sequencing strategies. Representative ongoing or completed clinical trials investigating the combination of ICIs with CT are summarized in Table 2.

**TABLE 2:** Clinical trials combining chemotherapy with immunotherapy.

Study	Year	Disease	CT	ICI	Schedule	Result	References
**Chemotherapy combing ICIs**							
NCT03215706	2021	NSCLC	Histology‐based platinum doublet chemotherapy (IV every 3 weeks for 2–4 cycles)	Nivolumab and ipilimumab	ICI concomitant CT	Median OS: 15.6 (experimental) vs. 10.9 (control) HR: 0.66 Serious treatment‐related AEs: 30% (exp) vs. 18% (ctrl)	[374]
NCT02039674	2016	NSCLC	Carboplatin(IV every 3 weeks × 4 cycles) and Pemetrexed (IV every 3 weeks × 4 cycles)	Pembrolizumab	ICI concomitant CT	ORR: 55% (pembro + chemo) vs. 29% (chemo alone) Grade ≥3 TRAEs: 39% vs. 26%	[375]
NCT02477826	2019	NSCLC	Histology‐based platinum‐doublet chemotherapy	Nivolumab and ipilimumab	ICI concomitant CT	Median OS 17.1 (NIVO + IPI) vs. 13.9 (chemo)	[376]
NCT00527735	2012	NSCLC	Paclitaxel and carboplatin	Ipilimumab	ICI within or 6 week later CT	Median PFS 5.7 vs. 5.5 vs. 4.6 (HR 0.69) Median OS 12.2 vs. 9.7 vs. 8.3 (HR 0.99)	[377]
NCT01473940	2019	Pancreatic cancer	Gemcitabine	Ipilimumab	ICI 90 min before CT	ORR: 14% (3/21 patients) Median PFS: 2.78 Median OS: 6.90	[378]
NCT03311789	2020	Biliary tract cancer (BTC)	Gemcitabine and cisplatin	Nivolumab	ICI 2 days after CTs	ORR: 55.6% Disease control rate (DCR): 92.6% Median PFS: 6.1 Median OS: 8.5	[379]
NCT02309177	2019	Pancreatic ductal adenocarcinoma	nab‐Paclitaxel and gemcitabine	Nivolumab	ICI concomitant CT	Median PFS: 5.5 median OS: 9.9	[380]
NCT02437370	2017	Urothelial cancer	Docetaxel or gemcitabine	Pembrolizumab	ICI concomitant CT	ORR: 33% Median PFS: 4.8; Grade 3+ adverse events in 54% of patients	[381]
NCT01681212	2015	Melanoma	Dacarbazine	Ipilimumab	ICI concomitant CT	ORR: 13%; study terminated early due to high‐grade liver toxicities	[382]
NCT02763579	2018	SCLC	Carboplatin plus etoposide	Atezolizumab and placebo	ICI concomitant CT	Median PFS 5.2 vs. 4.3 Median OS 12.3 vs. 10.3 The atezolizumab group showed a 13% higher survival rate at 12 and 18 months compared with placebo.	[383]
NCT02425891	2018	Advanced triple‐negative breast cancer (TNBC)	Nab‐paclitaxel and placebo	Atezolizumab	ICI concomitant CT	Median PFS 7.2 vs. 5.5 (HR 0.8) Median OS 21.3 vs. 17.6 (HR 0.84)	[384]
NCT02578680	2020	NSCLC (nonsquamous)	Pemetrexed + platinum (cisplatin or carboplatin)	Pembrolizumab	ICI concomitant CT	Median PFS 9.0 vs. 4.9 (HR 0.48) Median OS 22.0 vs. 10.7 (HR 0.56)	[33]
NCT02367781	2019	NSCLC (nonsquamous)	Carboplatin + nab‐paclitaxel	Atezolizumab	ICI concomitant CT	Median PFS 7.0 vs. 5.5 (HR 0·64) Median OS 18.6 vs. 13.9 (HR 0·79)	[385]
NCT03215706	2021	NSCLC	Histology dependent	Nivolumab + ipilimumab	ICI before CT	Median PFS 6.7 vs. 5.3 (HR 0.67) Median OS 15.6 vs. 10.9 (HR 0·66)	[386]
NCT03164616	2022	NSCLC	Histology dependent	Tremelimumab + durvalumab	ICI after CT	Median PFS 6.2 vs. 4.8 (HR 0.72) Median OS 14 vs. 11.7 (HR 0.77)	[387]
NCT03615326	2021	Gastric HER2+	Trastuzumab + 5‐FU + platinum or Capox	Nivolumab	ICI concomitant CT	Median PFS 74.4 vs. 51.9 Median OS ongoing	[388]
NCT02872116	2021	Gastric/GEJ/esophageal ADK HER2−	Capecitabine + oxaliplatin or Leucovorin + 5‐FU + oxaliplatin	Nivolumab	ICI concomitant CT	Median PFS 7.7 vs. 6.0 (HR 0·68) Median OS 14.4 vs. 11.1 (HR 0·71)	[389]
NCT03189719	2021	Esophageal	5‐FU + platinum	Pembrolizumab	ICI/3 weeks 5‐FU (1‐5d) + cisplatine (1 days)	Median PFS 6.3 vs. 5.8 (HR 0·65) Median OS 12.4 vs. 9.8 (HR 0·73)	[390]
NCT03875235	2024	Biliary tract cancers	Gemcitabine + cisplatin	Durvalumab	ICI concomitant CT	Median PFS (HR 0.75) Median OS 12.9 vs. 11.3 (HR 0.76)	[391]
**Chemotherapy combing other immunotherapies**							
NCT01740297	2016	Melanoma	T‐VEC	Ipilimumab	ICI 6 weeks later CT	ORR: 50% Durable response (≥6 months): 44% 18‐month PFS: 50% 18‐month OS: 67%	[392]
NCT00094653	2013	Melanoma	Peptide vaccine	Ipilimumab	ICI concomitant CT	No significant difference between ipilimumab + gp100 and ipilimumab alone (HR = 1.04; *p* = 0.76)	[393]
NCT01832870	2017	Prostate cancer	Sipuleucel‐T	Ipilimumab	ICI 1 week after CT	Acceptable tolerability	[394]
NCT00113984	2012	Prostate cancer	GVAX	Ipilimumab	ICI 15 d after CT	Well tolerated with sustained immune response and PSA control at 36 months	[395]
NCT02243371	2020	Pancreatic ductal adenocarcinoma	GVAX and CTX	Nivolumab	ICI 1 d before CT	Median PFS 2.27 vs. 2.23 (HR 0.90) Median OS 5.9 vs, 6.1 (HR 0.86,)	[396]
NCT02484404	2019	TNBC	Olaparib	Durvalumab	ICI concomitant CT	Partial response 44%; clinical benefit rate 67%; RP2D established with manageable toxicity	[397]
**Ongoing clinical trials**							
NCT03879512	2023	Relapsed high‐grade gliomas (HGG)	Cyclophosphamide	Nivolumab/ipilimumab	ICI concomitant CT	OS PFS Toxicity	/
NCT03349450	2024	Relapsed/refractory diffuse large B‐cell lymphoma	Cyclophosphamide	Pembrolizumab	ICI concomitant CT	OS 10.1 well tolerated	[398]
NCT03801304	2019	NSCLC	Vinorelbine	Atezolizumab	ICI (1/21 d) CT (3/weeks)	Acceptable tolerability	[399]
NCT03346642	2019	Diffuse large B‐cell lymphoma	Decitabine	PD‐1 antibody (SHR‐1210)	–	Unknown	/

/: no corresponding publication available; see www.clinicaltrials.gov for details.

## 5 The Safety and Toxicity Predictive Markers

Based on the clinical evidence outlined above, the combination of RT/CT and IT holds great promise for the future of cancer treatment. However, a key concern remains whether this combination therapy will lead to an increased incidence of adverse effects.

### 5.1 Overviews

In the context of combining RT with IT, in a prospective study of 16 patients who underwent combined stereotactic radiosurgery (SRS) and ipilimumab (10 mg/kg, treatment started 2 days after SRS), the combination was found to be safe, with no patients experiencing adverse effects greater than grade 3 [400]. Furthermore, a systematic review and meta‐analysis of 17 studies involving 534 patients showed that the 1‐year OS rate for patients with brain metastases treated with SRS in combination with ICIs was 64.6%, significantly higher than the 51.6% survival rate for those receiving nonconcurrent treatment (*p* < 0.001) and with the incidence rate of radiation necrosis was only 5.3%, which demonstrated that the combination of SRS and ICIs is not related to adverse effects [401]. Together with data from a series of trials [402, 403, 404], there is no conclusive evidence suggesting that RT combined with IT leads to a higher incidence of adverse reactions, and the toxicity remains within tolerable limits [405, 406].

In the context of combining CT with IT, a recent systematic analysis by Rached and colleagues [407], encompassing 20 RCTs, revealed that 18 of these studies reported a higher incidence of adverse events in patients receiving ICIs plus CT compared with CT alone. These findings suggest a potential synergistic toxicity between the two modalities, particularly in relation to gastrointestinal mucosal damage. Moreover, IT‐associated pneumonitis, when superimposed on CT‐induced pulmonary toxicity, may further exacerbate the severity of lung‐related complications.

Nevertheless, several studies have demonstrated that the toxicity profile of combined immunochemotherapy remains within an acceptable safety margin. The IMpower133 trial [408] showed no significant difference in overall adverse event rates between patients treated with atezolizumab plus CT (*n* = 198) and those receiving placebo plus CT (*n* = 196), despite a modest increase in immune‐related toxicities. Similarly, the CASPIAN trial [409] indicated that first‐line treatment with durvalumab in combination with platinum–etoposide CT for small‐cell lung cancer resulted in comparable rates of grade 3–4 treatment‐related adverse events (62 vs. 62%) and treatment‐related mortality (5 vs. 6%) relative to the control group. These data collectively suggest that the addition of PD‐L1 inhibitors to standard CT does not substantially increase the burden of severe toxicity and remains clinically manageable.

Despite limited conclusive evidence demonstrating that the combination of RT or CT with ICIs significantly increases the incidence of grade 3 or higher adverse events, the complexity of the resulting toxicity profile and its substantial inter‐individual and inter‐disease variability have raised considerable concern [410, 411]. The majority of current data are derived from retrospective, small‐scale, single‐center studies, which are inherently limited by retrospective bias in data collection, selection bias, and inconsistent definitions of toxicity [412, 413, 414]. These methodological constraints may compromise the reliability of the findings, particularly in smaller subgroups—such as patients with cutaneous versus noncutaneous primary tumors—where variability is even more pronounced [413]. To more accurately characterize the toxicity associated with combination therapy and enable precise risk stratification, future research must prioritize large‐scale, prospective, multicenter clinical trials with standardized criteria for toxicity assessment.

### 5.2 Mechanisms and Management

Both RT and CT can provoke toxic side effects, primarily through mechanisms involving tissue injury and immune activation. The damage in both normal and tumor cells is mainly arised from the DNA damage. When cells are exposed to ionizing radiation, their genomic DNA is directly affected by secondary electrons or indirectly by ROS, leading to irreversible DSBs and inflammation [415, 416, 417, 418]. The adverse effects associated with CT largely stem from its broad cytotoxicity, with commonly affected organs including the heart, lungs, and kidneys. Additional toxicities such as ototoxicity, peripheral neuropathy, and gastrointestinal disturbances are also frequently observed. Among these, cardiotoxicity is typically dose limiting and is most prominently associated with anthracyclines such as doxorubicin; pulmonary toxicity is more frequently observed in patients receiving bleomycin; and nephrotoxicity is closely linked to cisplatin administration [419]. ICIs, on the other hand, can provoke autoimmune‐like manifestations across multiple organ systems, known as immune‐related adverse events (irAEs) [420]. The infiltration of activated T cells into normal tissues appears to be a key driver of immune‐related toxicity, and recent evidence suggests it could also serve as a marker for irAEs [421, 422]. However, the precise pathological mechanisms underlying irAEs are still not fully understood. Different cell types exhibit varying toxic responses to RT [423, 424, 425]. Studies indicate that these reactions may depend on factors such as the differentiation state of cell, tissue residency, and the radiation dose and fractionation [426, 427, 428]. Consequently, the way RT and ICIs synergistically regulate immune responses may also influence the type and severity of treatment‐related adverse reactions. In the context of CT, elevated plasma levels of cardiac troponin I have been validated as a predictive biomarker for heightened susceptibility to anthracycline‐induced cardiotoxicity [429]. Moreover, distinct chemotherapeutic agents exert divergent effects on peripheral neurotoxicity, underscoring the heterogeneity of treatment‐associated adverse events [430].

Collectively, current evidence suggests that while severe toxicities such as pneumonitis may be potentially life‐threatening, most adverse events remain clinically manageable, with immune‐related toxicities emerging as particularly prominent. These findings underscore the critical need for personalized risk assessment and the development of proactive toxicity monitoring strategies to enhance the safety of combination therapies. In the combination of RT/CT and IT, toxicity management is a critical factor that influences both treatment efficacy and patient quality of life. Studies have shown that up to three‐quarters of patients receiving combined therapy may experience irAEs [431]. When detected early and properly managed, the incidence of irAEs decreases, demonstrating a correlation between irAEs and favorable prognosis [432, 433]. In the combination of RT/CT and IT, toxicity management is a critical factor that influences both treatment efficacy and patient quality of life. Studies have shown that up to three‐quarters of patients receiving combined therapy may experience irAEs [431]. When detected early and properly managed, the incidence of irAEs decreases, demonstrating a correlation between irAEs and favorable prognosis [432, 433]. The effective management of irAEs hinges on early recognition and timely intervention. To facilitate the implementation of personalized treatment strategies, patients should undergo comprehensive baseline assessments and diagnostic evaluations prior to initiating ICI therapy [434].

### 5.3 Predictive Biomarkers

Therefore, identifying and predicting biomarkers for toxicity risks is particularly important. However, the prediction, diagnosis, and characterization of irAEs remain challenging due to the complexity of the ICI mechanism, the unclear underlying mechanisms of irAEs, and other factors [435]. Studies have suggested that various cell‐related ratios (such as absolute lymphocyte count [436] and neutrophil/lymphocyte ratio [437]), thyroid‐stimulating hormone [438, 439], antibodies (such as antithyroglobulin antibodies) [438], cytokines (e.g., IFN‐γ [440], CXCL9/10/11 [441]), tumor tissue markers [442], the microbiome [443, 444], and even genetic characteristics [445, 446] are all associated with the occurrence of adverse events. A prospective study by Azria and colleagues [447] demonstrated that a combination of low levels of radiation‐induced CD8⁺ T cell apoptosis and the presence of at least four radiation‐sensitivity‐associated single nucleotide polymorphisms in candidate genes—including ATM, SOD2, XRCC1, XRCC3, TGFB1, and RAD21—may serve as a potential biomarker for predicting the risk of severe late toxicities in patients undergoing RT. In parallel, Chaouni et al. [448] investigated predictive markers for cutaneous adverse effects following radiation therapy. Furthermore, pioneering work by Weidhaas and colleagues [449] revealed that germline variations within microRNA regulatory pathways can act as cross‐cancer biomarkers to predict irAEs associated with IT—independently of treatment efficacy. With the rapid evolution of artificial intelligence, radiomics‐based prediction models now allow for the extraction of IT response‐related features from standard CT imaging. These noninvasive and generalizable biomarkers have shown promising predictive performance, as evidenced by Trebeschi et al. [450], who reported high predictive accuracy in NSCLC (AUC up to 0.83, *p* < 0.001) and moderate performance in melanoma (AUC = 0.64, *p* = 0.05). Notably, these features were significantly enriched in mitosis‐related gene pathways, suggesting that increased proliferative potential may underlie enhanced responsiveness to IT.

Despite this, research on toxicity prediction in the context of combined RT/CT and ICI therapy is still limited, and identifying ideal RT/CT–IT cotoxicity biomarkers remains an area that is not yet fully explored. Future research should focus on further verifying and integrating multiple genomic markers to establish a more comprehensive toxicity prediction model. This approach would help in promoting individualized toxicity management and optimizing the effects of combined RT–IT treatment.

## 6 Conclusion

This review highlights the immune effects induced by RT/CT and the specific mechanisms involved. It describes the different RT/CT doses and fractionation that lead to varying changes in immune microenvironments, the sequential order of IT and RT/CT, and the safety considerations of combining the two approaches.

With rapid advancements in cancer treatment, novel therapeutic approaches are continuously emerging. In terms of immune stimulation, particle‐based RT (PRT) is becoming to serve as an effective alternative to photon‐based RT and standing out as a promising new combination to IT [30]. Due to its higher linear energy transfer (LET) and denser ionization capabilities, PRT can produce more complex and irreparable DNA DSBs, a higher relative biological effectiveness, and better preservation of normal tissue than photon RT [451, 452, 453, 454]. Evidence suggests that PRT may induce stronger immunogenicity than photon radiation. In a study conducted by Yoshimoto et al. [455], researchers measured levels of HMGB1 in samples exposed to equivalent doses of PRT and photon RT 72 h posttreatment, showing that HMGB1 levels were comparable. More interestingly, the LET of PRT reached 50 keV/µm, which means more carbon ions concentration and higher energy release directly into tumor tissue, effectively damaging tumor cell DNA and enhancing therapeutic efficacy [456]. Similarly, studies indicate that PRT may cause more persistent DNA damage, genomic mutations or instability, potentially resulting in higher antigen release and increased diversity within the T‐cell repertoire compared with photons RT [457, 458, 459]. In summary, PRT shows potential for enhanced synergy with IT and initial studies [460] have explored its combination with immune‐based treatments. Its future application needs further attention and investigation.

Several CT drugs encapsulated using nanodelivery strategies have been clinically approved for cancer treatment [461]. Compared with conventional formulations, these nanocarrier systems significantly enhance in vivo stability, prolong circulation time, and enable precise targeting [462, 463]. Technologies such as liposomes and polymeric nanoparticles have improved therapeutic efficacy while reducing toxicity. The “free drug plus nano‐platform” approach has become the mainstream application [464, 465, 466]. Lim et al. [467] developed two types of poly(lactic‐co‐glycolic acid)‐based nanoparticles loaded with the chemotherapeutic agent PTX and immune modulators: one containing CpG oligodeoxynucleotides (PCN) to activate bone marrow‐derived DCs. Compared with PTX alone, sequential administration of PCNs and PINs significantly enhanced antitumor activity and prolonged survival in B16‐F10 melanoma‐bearing mice (*p* < 0.05). Additionally, Li et al. [468] designed TME‐responsive prodrug vesicles codelivering oxaliplatin prodrug and a photosensitizer to induce ICD and stimulate antitumor immunity. When combined with CD47 blockade antibody (αCD47), this approach markedly suppressed tumor growth, metastasis, and recurrence, highlighting the promising potential of nanoplatforms in chemo‐IT combinations.

For IT, a new generation of ICBs is also entering clinical practice, with the potential to enhance the effects of RT. T‐cell immunoglobulin (Ig) and mucin domain 3 (TIM‐3) (encoded by Havcr2) is an Ig and mucin domain‐containing cell surface molecule [469], and it is a highly expressed marker on functionally exhausted T cells within the immune‐suppressive TME [470]. The combination of anti‐TIM3 therapy with RT has shown promising results. In a study by Kim et al. [471], a triple combination of TIM‐3, PD‐1, and SRS not only demonstrated the best survival rates but also increased the CD8α/Treg cell ratio and IFN‐γ secretion compared with individual or dual combinations. Kim et al. [472] reached similar conclusions in their research. de Mingo Pulido et al. [473] demonstrated in a murine breast cancer model that TIM‐3 expression on tumor‐associated DCs restricts CT‐induced immune activation. Combination treatment with PTX and anti‐TIM‐3 antibody significantly enhanced CD8⁺ T cell effector functions, leading to marked tumor growth inhibition without evident toxicity. This synergistic effect depended on CD8⁺ T cell activity, as reflected by increased granzyme B (GZMB) expression during combination therapy, suggesting a positive modulation of the tumor immune microenvironment by chemo‐IT. These findings hold clinical significance and warrant further validation in clinical studies. Combining IT with tumor‐targeted agents to support radio/chemo‐IT represents a new treatment strategy. In a research by Li et al. [474], combining anti‐HER2 and anti‐CD47 therapy in both in vivo and in vitro models of ovarian cancer (SKOV3) and breast cancer (BT474) enhanced antitumor activity, reduced the toxicity, and extended survival. It also is a promising approach to further enhance the efficacy of radio/chemo‐IT.

In summary, the mechanistic insights, preclinical investigations, and clinical evidence presented herein collectively support the premise that successful integration of RT/ CT with IT necessitates the adaptive modulation of traditional RT/CT parameters to effectively engage the immune response. Specifically, factors such as RT dose and fractionation schedules, the selection and regulation of chemotherapeutic agents, as well as the timing of combined RT, CT, and IT administration, are critical determinants influencing the magnitude of antitumor immune activation. Concurrently, advancements in RT technologies alongside emerging CT strategies—leveraging nanodelivery systems and immune regulatory targets—offer promising new avenues for the evolution of immuno‐adaptive chemoradiotherapy. Looking ahead, the identification and validation of biomarkers associated with therapeutic efficacy and toxicity hold significant potential to refine the management of iAEs, thereby enhancing both the safety profile and clinical outcomes of combined chemoradiotherapy and IT regimens.

## Author Contributions

Xinmin Wang and Jing Jing contributed to conception and manuscript design. Xinmin Wang wrote the manuscript. Xinmin Wang and Jing Jing were involved in manuscript revision. The authors read and approved the final manuscript.

## Ethics Statement

The authors have nothing to report.

## Conflicts of Interest

The authors declare that the research was conducted in the absence of any commercial or financial relationships that could be construed as a potential conflict of interest.

## Data Availability

Data sharing not applicable to this article as no datasets were generated or analyzed during the current study. All data and information in this review can be found in the reference list.
